# Therapeutic depletion of CCR8^+^ tumor-infiltrating regulatory T cells elicits antitumor immunity and synergizes with anti-PD-1 therapy

**DOI:** 10.1136/jitc-2020-001749

**Published:** 2021-02-15

**Authors:** Helena Van Damme, Bruno Dombrecht, Máté Kiss, Heleen Roose, Elizabeth Allen, Eva Van Overmeire, Daliya Kancheva, Liesbet Martens, Aleksandar Murgaski, Pauline Madeleine Rachel Bardet, Gillian Blancke, Maude Jans, Evangelia Bolli, Maria Solange Martins, Yvon Elkrim, James Dooley, Louis Boon, Julia Katharina Schwarze, Frank Tacke, Kiavash Movahedi, Niels Vandamme, Bart Neyns, Sebahat Ocak, Isabelle Scheyltjens, Lars Vereecke, Frank Aboubakar Nana, Pascal Merchiers, Damya Laoui, Jo Agnes Van Ginderachter

**Affiliations:** 1 Laboratory of Cellular and Molecular Immunology, Vrije Universiteit Brussel, Brussels, Belgium; 2 Myeloid Cell Immunology Lab, VIB Center for Inflammation Research, Brussels, Belgium; 3 VIB Discovery Sciences, VIB, Ghent, Belgium; 4 Oncurious NV, Leuven, Belgium; 5 VIB-UGent Center for Inflammation Research, VIB, Ghent, Belgium; 6 Department of Biomedical Molecular Biology, Faculty of Science, Ghent University, Ghent, Belgium; 7 Department of Internal Medicine and Pediatrics, Ghent University, Ghent, Belgium; 8 Host-Microbiota-Interaction Lab (HMI), VIB-UGent Center for Inflammation Research, Ghent, Belgium; 9 Laboratory of Lymphocyte Signalling and Development, Babraham Institute, Cambridge, Cambridgeshire, UK; 10 Polpharma Biologics, Utrecht, The Netherlands; 11 Department of Medical Oncology, UZ Brussel, Brussels, Belgium; 12 Department of Medicine III, RWTH Aachen University, Aachen, Nordrhein-Westfalen, Germany; 13 Data Mining and Modelling for Biomedicine, VIB-UGent Center for Inflammation Research, Ghent, Belgium; 14 Department of Applied Mathematics, Computer Science and Statistics, Ghent University, Ghent, Belgium; 15 Institut de Recherche Expérimentale et Clinique (IREC), Pôle de Pneumologie, ORL et Dermatologie (PNEU), UCLouvain, Louvain-la-Neuve, Belgium; 16 Division of Pneumology, CHU UCL Namur, Yvoir, Namur, Belgium; 17 Division of Pneumology, Cliniques universitaires Saint-Luc, Brussels, Belgium

**Keywords:** biomarkers, tumor, immunotherapy, lymphocytes, tumor-infiltrating, receptors, immunologic

## Abstract

**Background:**

Modulation and depletion strategies of regulatory T cells (Tregs) constitute valid approaches in antitumor immunotherapy but suffer from severe adverse effects due to their lack of selectivity for the tumor-infiltrating (ti-)Treg population, indicating the need for a ti-Treg specific biomarker.

**Methods:**

We employed single-cell RNA-sequencing in a mouse model of non-small cell lung carcinoma (NSCLC) to obtain a comprehensive overview of the tumor-infiltrating T-cell compartment, with a focus on ti-Treg subpopulations. These findings were validated by flow cytometric analysis of both mouse (LLC-OVA, MC38 and B16-OVA) and human (NSCLC and melanoma) tumor samples. We generated two CCR8-specific nanobodies (Nbs) that recognize distinct epitopes on the CCR8 extracellular domain. These Nbs were formulated as tetravalent Nb-Fc fusion proteins for optimal CCR8 binding and blocking, containing either an antibody-dependent cell-mediated cytotoxicity (ADCC)-deficient or an ADCC-prone Fc region. The therapeutic use of these Nb-Fc fusion proteins was evaluated, either as monotherapy or as combination therapy with anti-programmed cell death protein-1 (anti-PD-1), in both the LLC-OVA and MC38 mouse models.

**Results:**

We were able to discern two ti-Treg populations, one of which is characterized by the unique expression of *Ccr8* in conjunction with Treg activation markers. *Ccr8* is also expressed by dysfunctional CD4^+^ and CD8^+^ T cells, but the CCR8 protein was only prominent on the highly activated and strongly T-cell suppressive ti-Treg subpopulation of mouse and human tumors, with no major CCR8-positivity found on peripheral Tregs. CCR8 expression resulted from TCR-mediated Treg triggering in an NF-κB-dependent fashion, but was not essential for the recruitment, activation nor suppressive capacity of these cells. While treatment of tumor-bearing mice with a blocking ADCC-deficient Nb-Fc did not influence tumor growth, ADCC-prone Nb-Fc elicited antitumor immunity and reduced tumor growth in synergy with anti-PD-1 therapy. Importantly, ADCC-prone Nb-Fc specifically depleted ti-Tregs in a natural killer (NK) cell-dependent fashion without affecting peripheral Tregs.

**Conclusions:**

Collectively, our findings highlight the efficacy and safety of targeting CCR8 for the depletion of tumor-promoting ti-Tregs in combination with anti-PD-1 therapy.

## Background

Regulatory T cells (Tregs) are an immunosuppressive subset of T lymphocytes known to play a crucial role in immune homeostasis and self-tolerance.^1^ They are characterized by the expression of the Foxp3 transcription factor, which is known to be a master regulator of Treg differentiation and suppressor function.^1^ Several mechanisms have been reported through which Tregs can mediate their suppressive function, including the release of immunosuppressive cytokines such as interleukin (IL)-10 and TGF-β (transforming growth factor-beta), the sequestering of IL-2 via the constitutive and high expression of CD25 and the cell-surface expression of immune checkpoint molecules such as CTLA-4, PD-1 and LAG-3.^2^

In the tumor microenvironment (TME), Tregs (tumor-infiltrating Tregs or ti-Tregs) have been shown to contribute to the suppression of antitumor immune responses and the development of an immunosuppressive TME in distinct cancer types.^3 4^ This protumoral role of Tregs is corroborated by the finding that a high Foxp3^+^ Treg infiltration correlates with a poor prognosis in multiple cancer types.^5 6^ Moreover, Tregs have been shown to hamper the efficacy of immunotherapy,^7^ emphasizing the need for potent and specific ti-Treg targeting strategies.

Several Treg-modulating and Treg-depleting strategies have been shown to reduce tumor burden and increase the antitumor immune response in both experimental models and the clinic.^8–16^ However, despite their apparent therapeutic benefit, most of these strategies have severe drawbacks that need to be taken into consideration. Most importantly, the systemic depletion of Tregs can result in severe autoimmunity.^5^ Furthermore, current strategies mainly target molecules that are expressed on both Tregs and effector T cells, including CD25^9 12 17^ and CTLA-4,^8 10^ resulting in the potential ablation of tumor-reactive T cells. These findings highlight the need for a highly selective marker which allows the specific targeting of ti-Tregs, without affecting effector T cells and peripheral Tregs.

Several recent studies have determined the transcriptome of ti-Tregs in distinct types of human cancer, including breast, hepatocellular, colorectal and non-small cell lung carcinoma (NSCLC) as well as (metastatic) melanoma,^18–23^ showing the consistent upregulation of the *CCR8* gene.^18–22^ The CC chemokine receptor CCR8 is a seven transmembrane G-protein coupled receptor (GPCR) with a high affinity for human/mouse CCL1, mouse CCL8 (mCCL8) and human CCL18 (hCCL18), the latter of which is a functional analog of mCCL8.^24^ Interestingly, in patients with breast and pancreatic cancer, high CCR8^+^ Treg numbers correlated with more advanced stages of the disease and a decreased overall survival.^20 25^ However, multiple questions remain as to the nature of CCR8^+^ Tregs in the TME, the regulation of CCR8 expression and whether it is functionally involved in ti-Treg activity (providing the rationale for CCR8 blockade) or should merely be considered as a biomarker for ti-Tregs (providing the rationale for CCR8 targeting and ti-Treg depletion).

To provide insight into these matters, we employed single-cell RNA-sequencing on the tumor T-cell infiltrate and identified two main ti-Treg subsets, one of which showing enhanced expression of CCR8 and various activation markers and displaying increased T-cell suppressive acitivity. CCR8 expression is the result of TCR-mediated Treg activation, but is not crucial for the recruitment, activation or suppressive capacity of these cells. Hence, selective natural killer (NK) cell-mediated depletion of the CCR8^+^ ti-Tregs using newly generated anti-CCR8 nanobody-Fc fusions, caused a significant reduction in tumor growth and synergized with anti-PD-1 therapy, resulting in complete tumor remission and immunological memory. However, CCR8 blockade alone without simultaneous Treg depletion was not sufficient to show antitumor effects.

## Materials and methods

### Mouse strains

Female C57BL/6 mice were purchased from Janvier. Ccr8^-/-^ (C57BL/6) and Foxp3^Thy1.1^ (C57BL/6) mice were kindly provided by Frank Tacke (Aachen University, Germany) and Adrian Liston (KULeuven, Belgium), respectively. OT-II (B6.Cg-Tg(TcraTcrb)425Cbn/J) mice were purchased from Charles River. Ccr8^-/-^ and Foxp3^Thy1.1^ mice were crossed to generate the Ccr8^−/−^ Foxp3^Thy1.1^ C57BL/6 mice.

### Cell cultures and tumor models

The LLC-OVA, MC38 and B16-OVA cell lines were kindly provided by Dmitry Gabrilovich (The Wistar Institute, Philadelphia, USA), Massimiliano Mazzone (VIB-KULeuven, Belgium) and Karine Breckpot (Vrije Universiteit Brussel, Belgium), respectively. These cell lines and ex vivo culture of splenocytes and T cells were maintained as previously described.^26^ The LLC-OVA cells were harvested and resuspended to a concentration of 3×10^6^ cells/200 µl HBSS, which was subsequently injected into the right flank of syngeneic 6 to 12 week-old female C57BL/6 mice. Tumor volume was determined via caliper measurements and was calculated via the following formula: Volume = *π* × (*d*^2^ ×*D*)/6, where *d* symbolizes the minor tumor axis and *D* symbolizes the major tumor axis. In the case of anti-CCR8 Nb-Fc or anti-PD-1 monoclonal Ab (mAb) treatment, tumor-bearing mice were injected intraperitoneally with 200 µg of the respective nanobody/Antibody or InVivoMab rat IgG2a isotype control Abs (clone 2A3) (Bio X Cell) in 100 µl of HBSS. A mouse IgG1-D265A Fc-silenced version of anti-mouse PD-1 mAb RMP1-14 was used that more closely reflects the mode of action of clinical anti-PD-1 mAbs (Absolute Antibody, catalog # Ab00813-1.32).^27 28^

### In vivo cell depletion

NK cells were depleted during LLC-OVA tumor growth by intraperitoneal injection of 300 µg of anti-NK1.1 (PK136), 2 days prior to the administration of isotype or anti-CCR8 Nb-Fc (online supplemental figure S10A). Neutrophils were depleted by alternating (24 hours) intraperitoneal injections of 75 µg of anti-Ly6G (1A8) and 150 µg of mouse anti-rat (MAR18.5),^29^ 4 days prior to the administration of anti-CCR8 Nb-Fc (online supplemental figure S10A). Macrophages were depleted by feeding the mice chow containing the highly selective CSF-1R inhibitor PLX5622, starting 5 days prior to the administration of anti-CCR8 Nb-Fc (online supplemental figure S10A). The other treatment groups received control chow.

10.1136/jitc-2020-001749.supp1 Supplementary data

### In silico analysis of TCGA data

Human data was recovered from The Cancer Genome Atlas (TCGA) (available at http://cancergenome.nih.gov) and analyzed using R2 (available at http://r2.amc.nl), a genomics analysis and visualization platform, and GraphPad Prism 8.0 software.

### Lung cancer and melanoma patient samples

Blood samples and fresh tumor tissue were obtained from 11 healthy donors and from 11 patients, respectively, undergoing surgical resection at three Belgian hospitals: UZ Brussel (melanoma samples) and CUSL and CHU UCL Namur (NSCLC samples and healthy donors). Clinical patient information can be found in online supplemental table S2. After resection, samples were immediately transported on ice to the research facilities at the Vrije Universiteit Brussel. Human tumor single cell suspensions were prepared as previously described.^26^

### Ex vivo single-cell preparation, flow cytometry, cell sorting

Preparation of single cell suspensions, subsequent flow cytometric analysis and/or cell sorting is described in detail in the online supplemental materials and methods.

### Single cell RNA sequencing using the 10x Genomics platform

Single-cell suspensions derived from the tumor were obtained using the above-mentioned procedures. For the analysis of the T-cell infiltrate, we pooled five tumors grown for 12 days in wild type (WT) C57BL/6 mice. The single cell suspensions were stained with APC-Cy7-labeled anti-CD45, PeCy7-labeled anti-CD11b, FITC-labeled anti-TCRβ and DAPI. Subsequently, approximately 50,000 live CD45^+^CD11b^−^TCRβ^+^ cells were sorted into ME medium using the BD FACSAria III (BD Biosciences). The sorted cells were subsequently centrifuged and resuspended in PBS (phosphate buffered saline) +0.04% bovine serum albumin at room temperature at an estimated final concentration of 1000 cells/µl. Next, the single-cell Bead-in Emulsions and single-cell RNA-sequencing (scRNA-seq) library was prepared as previously described.^30^ The scRNA-seq experiment was performed once, giving rise to a scRNA-seq libraries encompassing approximately 2730 single cells. The obtained sequencing library was then sequenced as previously described,^30^ the average of the mean reads per cell across was >32,000, with an average sequencing saturation metric of >68%. The gene expression matrices were preprocessed and filtered using the SCRAN and Scater R packages.^31^ The detection of outlier cells, generation of PCA plots, removal of low-abundance genes, normalization and unsupervised clustering were performed as previously described.^30^ The obtained clustering was visualized in two-dimensional scatter plots via Uniform Manifold Approximation and Projection (UMAP) using the Seurat V3.0 R package.

### Single-cell regulatory network inference and clustering using pySCENIC

We performed pySCENIC by starting from the filtered raw counts and following the proposed workflow using the latest Singularity image. The pySCENIC output (loom file) was then analyzed using the SCopeLoomR R package. We created a binary heatmap indicating if a regulon is active or not in a particular cell, based on the output area under the recovery curve (AUC) values and AUC thresholds. Besides plotting the binary values, we also transformed these AUC values into values ranging from 0 to 1 for each regulon and plotted this in a heatmap with a blue–red color scale. The aheatmap function of the NMF R package was used for creating the heatmap and the regulons are clustered hierarchically.

### RNA extraction and cDNA preparation for qPCR

RNA extraction and subsequent qPCR reaction were performed as previously described,^26^ a detailed description of the procedures can be found in the online supplemental materials and methods.

### Suppression assays and OT-II T cell activation

The suppression assays and OT-II T cell activation were performed as previously described,^26^ a detailed description of the procedures can be found in the online supplemental materials and methods.

### Adoptive Treg transfer

Splenic (CD4^+^CD25^+^) Tregs were isolated from both WT C57BL/6 and OT-II spleens as described in the online supplemental materials and methods. The obtained Tregs were labeled with CellTrace Violet (Thermo Fisher Scientific) and subsequently resuspended in HBSS to a final concentration of 6×10^5^ cells/20 µl. Of this single cell suspension, 20 µl was injected directly into the tumor of day 9 LLC or LLC-OVA subcutaneously tumor-bearing mice. After 48 hours, the tumors from each group were collected and processed to a single cell suspension. Using flow cytometry, the CellTrace^+^ cells within the TME were evaluated for their upregulation of CCR8 expression.

### Immunizations and phage display selections

Nanobodies were generated through immunization of llamas and alpacas with DNA as previously described.^32 33^ A brief description of the procedures can be found in the online supplemental materials and methods.

### Screening of CCR8 selection outputs

Nb clones from the mouse CCR8 immunization and selection campaign, were screened by means of flow cytometry for binding to HEK293 cells that were transiently transfected with full length mouse CCR8 or with N-terminal deletion mouse CCR8 (delta16-3XHA) plasmid DNA (online supplemental table S1), in comparison to mock transfected control cells. A detailed description of the procedures can be found in the online supplemental materials and methods.

### Cloning, expression and purification of Nb-Fc fusions

Tetravalent Nb-Fc constructs were generated combining a copy each of Nb-I and Nb-II per Fc arm separated by a 10 amino acid flexible GlySer linker and fused by a second 10 amino acid flexible GlySer linker to the hinge region of the mouse IgG2a Fc domain. A detailed description of the generation of these constructs can be found in the online supplemental materials and methods.

### Binding and competition experiments, cAMP HTRF assay for Gi-coupled receptors and apoptosis assay

A detailed description of the procedures used to characterize the nanobody constructs can be found in the online supplemental materials and methods.

### Histology

A detailed description of the procedures used for histological analysis of colon and ileum tissues can be found in the online supplemental materials and methods.

### FITC–dextran intestinal permeability assay

A detailed description of the procedures used to characterize intestinal permeability can be found in the online supplemental materials and methods.

### Statistics

All graphs show mean±SEM. Statistical significance (p value <0.05) was determined in GraphPad Prism 8.0 software. For relevant pairwise comparisons, either paired or unpaired Student’s t-tests were performed. For the comparison of multiple groups, one-way analysis of variance (ANOVA) was performed, followed by a post-test. Tumor growth curves were compared by two-way ANOVA with Holm-Sidak multiple comparisons test. To assess correlation, a Pearson correlation coefficient was calculated. For statistically significant differences, the p value is indicated in graphs as the following: *p<0.05, **p<0.01, ***p<0.001, ****p<0.0001.

## Results

### Expression of *Ccr8* is detected in three distinct tumor-infiltrating T-cell subpopulations

**Figure 1:**
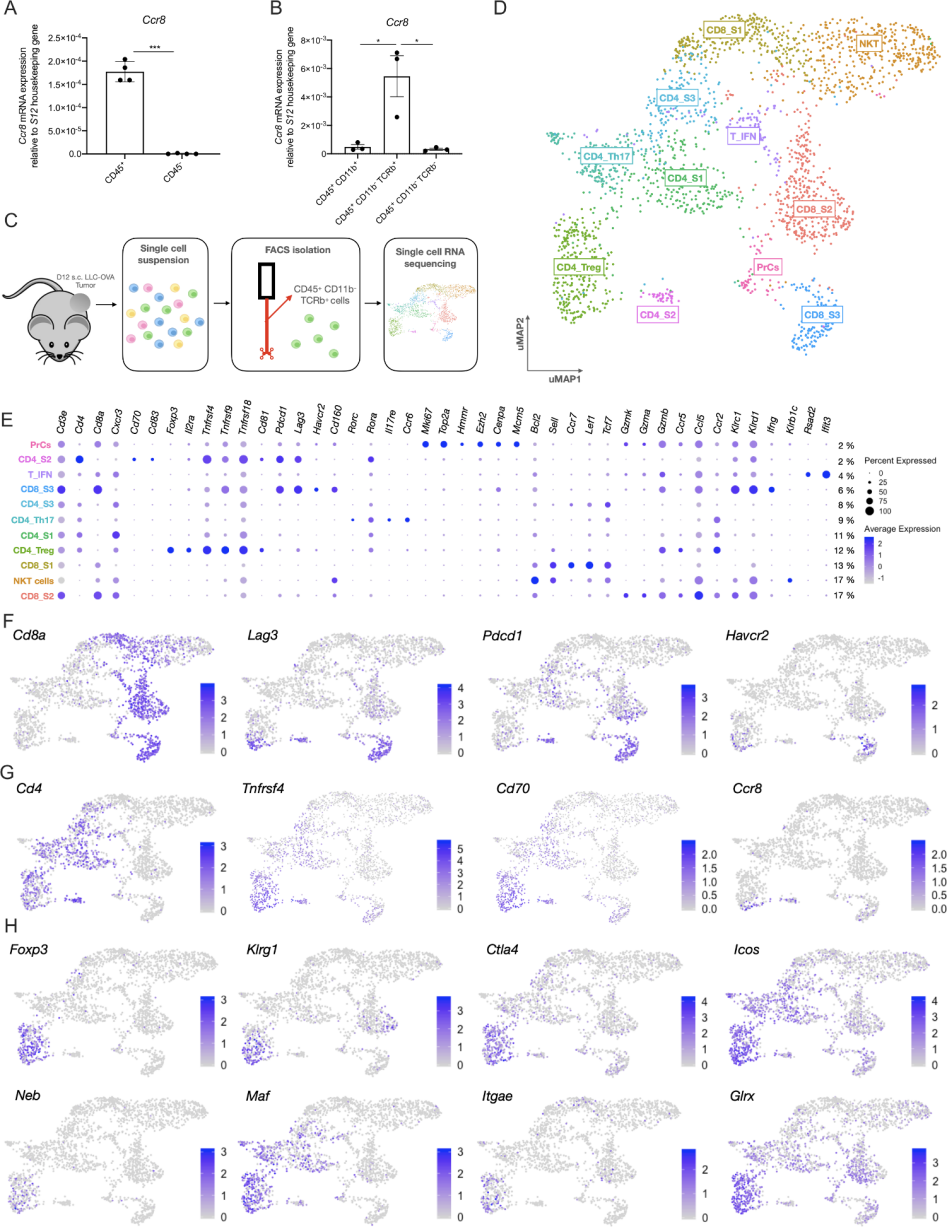
Single cell RNA-seq analysis of the LLC-OVA tumor-infiltrating T-cell compartment. (A) *Ccr8* expression in the CD45^+^ hematopoietic cell subset and CD45^−^ non-hematopoietic cell subset of LLC-OVA tumors measured via qRT-PCR (n=4). (B) *Ccr8* expression in the CD45^+^CD11b^+^, CD45^+^CD11b^−^TCRβ^+^ and CD45^−^CD11b^−^TCRβ^−^ cell subsets measured via qRT-PCR (n=3). (C) Schematic overview of the single cell RNA-seq experiment. (D) UMAP plot of 2.603 WT NKT/T cells isolated from s.c. LLC-OVA tumors revealing the presence of 11 T-cell subsets. (E) Dot plot showing the relative gene expression of several signature genes within the distinct T-cell subsets. The size of the dots relates to the % positive cells within each T-cell population. The color code relates to the relative expression level of the gene. (F) UMAP plots showing expression of several key marker genes of the CD8_S3 subset. (G) UMAP plots showing expression of several key marker genes of the CD4_S2 subset. (H) UMAP plots showing expression of several key marker genes of the CD4_Treg subset. (A, B) Data shown as mean±SEM. (A) ***p<0.001 by paired Student’s t-test, (B) *p<0.05 by one-way ANOVA. ANOVA, analysis of variance; LLC, Lewis Lung Carcinoma; mRNA, messenger RNA; NK, natural killer; s.c., subcutaneously; Treg, regulatory T cell; UMAP, Uniform Manifold Approximation and Projection; WT, wild type.

To obtain an idea about the expression pattern of *Ccr8* in the TME, *Ccr8* expression was first assessed via qRT-PCR within the CD45^+^ hematopoietic and CD45^−^ non-hematopoietic fraction of subcutaneously grown Lewis Lung Carcinoma (LLC)-OVA, B16-OVA melanoma and MC38 colon carcinoma tumors. *Ccr8* expression was restricted to the CD45^+^ hematopoietic cells (figure 1A, online supplemental figure S1A), and more specifically to the CD11b^−^TCRβ^+^ T/NKT-cell compartment (figure 1B). To further identify the *Ccr8*-expressing cells within the CD45^+^CD11b^−^TCRβ^+^ compartment of LLC-OVA tumors, we relied on single-cell RNA-sequencing using the 10x chromium platform (figure 1C). Unsupervised clustering, dimensionality reduction and UMAP projections were performed on 2.603 tumor-infiltrating T/NKT cells (figure 1D) and individual clusters were identified based on their expression of known marker genes (figure 1E). Among the CD8^+^ T cells, the CD8_S1 cluster showed high expression levels of *Bcl2*, *Sell* (CD62L), *Tcf7* and *Lef1* (figure 1E, online supplemental figure S1B), indicative of a memory CD8^+^ T cell phenotype.^34–36^ The CD8_S2 cluster represented the largest fraction of tumor-infiltrating CD8^+^ T cells (figure 1D) and was marked by the expression of several genes associated with effector CD8^+^ T cells (*Gzmk*, *Gzma*, *Gzmb, Klrc1*, *Klrd1, Ccr5* and *Ccr2*)^37^ (figure 1E, online supplemental figure S1B). Finally, the CD8_S3 cluster also showed a highly activated CD8^+^ T cell phenotype, but with the additional elevated expression of *Ifng*, *Tnfrsf4* (OX-40, CD134), *Tnfrsf9* (4-1BB, CD137) and several immune checkpoint molecules such as *Pdcd1* (PD-1), *Lag3* (CD223), *Havcr2* (TIM-3) and *Cd160*, indicative of a dysfunctional CD8^+^ T-cell phenotype (figure 1E, F, online supplemental figure S1B)^38^. Interestingly, *Ccr8* expression could be detected in a fraction of these dysfunctional CD8^+^ T cells (figure 1G). *Ccr8* was also expressed in the CD4_S2 cluster, which showed high expression levels of *Pdcd1*, *Lag3* and *Tnfrsf4*, but also of the activation markers *Cd70* and *Cd83* (figure 1E–G), suggesting that these are dysfunctional CD4^+^ T cells. Other CD4^+^ T cell clusters included the naive CD4_S1 cluster (high expression of *Cd4* and *Cxcr3*), the CD4_Th17 cluster (high expression of *Il17re*, *Ccr6, Rorc* and *Rora*) and cluster CD4_S3 (increased expression of *Il7r*, *Socs3*, *Asap1* and *Tcf7*) (figure 1E, online supplemental figure S1B), all of which were *Ccr8*-negative. Conversely, CD4^+^ regulatory T cells (CD4_Treg), identified based on their high expression of Treg-associated genes such as *Foxp3* (figure 1E, H and online supplemental figure S1B), *Klrg1*, *Ctla4* (figure 1H), *Tnfrsf4*, *Tnfrsf9*, *Tnfrsf18* (GITR), *Cd81*, *Il2ra* (CD25), *Icos* (CD278) and *Il10* (figure 1E, H), were partly *Ccr8*-positive, indicating that there might be distinct Treg subsets present within the TME (figure 1G). Finally, *Ccr8* expression was negative in cluster T_IFN (increased expression levels of multiple interferon-induced genes such as *Rsad2*, *Ifit3* (figure 1E), *Ifit3*, *Ifit*1 and *Isg15* (online supplemental figure S1B)), cluster PrCs (high expression of cell cycle genes, including *Mki67, Top2a, Hmmr, Ezh2, Cenpa* and *Mcm5* (figure 1E, and online supplemental figure S1B)) and cluster NKT (expression of NK-associated genes such as *Klrb1c* (NK1.1, figure 1E), *Klra7*, *Klra6* and *Klre1* (online supplemental figure S1B)).

Overall, these data show that the expression of *Ccr8* within the LLC-OVA tumor immune compartment is restricted to a subset of Tregs, but also to populations of dysfunctional CD8^+^ and CD4^+^ T cells.

### scRNA-seq analysis reveals the presence of two main tumor-infiltrating Treg populations, one of which expresses *Ccr8*

**Figure 2:**
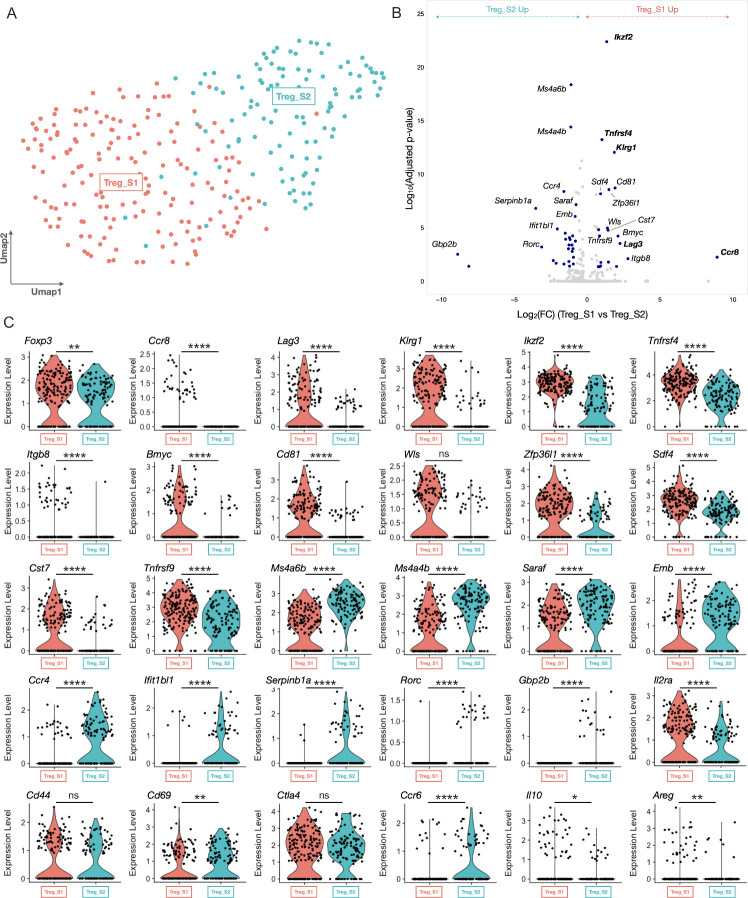
Single cell RNA-seq analysis of the ti-Tregs. (A) UMAP plot of 289 ti-Tregs isolated from s.c. LLC-OVA tumors revealing the existence of two ti-Treg subsets. (B) Volcano plot showing the genes that are differentially expressed between Treg_S1 and Treg_S2. (C) Violin plots showing expression of several key marker genes of the Tregs_S1 (red) and Treg_S2 (turquoise) subsets. (C) *p<0.05, **p<0.01 and ****p<0.0001 by unpaired Student’s t-test. LLC, Lewis Lung Carcinoma; s.c., subcutaneously; ti-Treg, tumor-infiltrating regulatory T cell; UMAP, Uniform Manifold Approximation and Projection.

To obtain a more detailed insight in the heterogeneity of the Treg compartment, 289 cells belonging to the CD4_Treg cluster were re-clustered, revealing two distinct Treg subsets (Treg_S1 and Treg_S2, figure 2A, B). Both clusters showed expression of *Foxp3*, though this Treg master regulator gene was significantly higher expressed in Treg_S1 (figure 2C). Moreover, the Treg_S1 subset showed higher expression levels of several activation markers and immune checkpoint molecules, including *Lag3*, *Klrg1*, *Tnfrsf4*, *Tnfrsf9* and *Il2ra,* but also of transcription factor *Ikzf2* (Helios), co-stimulatory molecule *Cd81* and secretory molecules *Areg* and *Il10* (figure 2B, C), indicative of a highly suppressive phenotype. Aside from these well-defined Treg markers, Treg_S1 also showed significantly higher expression of *Itgb8*, *Bmyc*, *Cst7*, *Wls* and *Sdf4* (figure 2B, C). Finally, *Ccr8* was mainly expressed in this highly activated Treg_S1 subset (figure 2B, C).

Conversely, the Treg_S2 subset was characterized by a significantly higher expression of *Saraf*, *Emb*, *Serpinb1a*, *Ifit1bl1, Gbp2b* and the GITR-associated membrane adaptors *Ms4a6b* and *Ms4a4b.*^39^ Interestingly, Treg_S2 also showed significantly higher expression levels of the transcription factor *Rorc* and chemokine receptors *Ccr4* and *Ccr6* (figure 2B, C), together suggesting a more Th17-like Treg phenotype, as has been previously described.^40^ Other well-known T-cell activation genes such as *Cd44* and *Ctla4* showed similar expression levels in both Treg subsets (figure 2C).

### The CCR8 protein is predominantly upregulated by the highly activated and strongly suppressive Treg subpopulation in both mouse and human tumors

**Figure 3:**
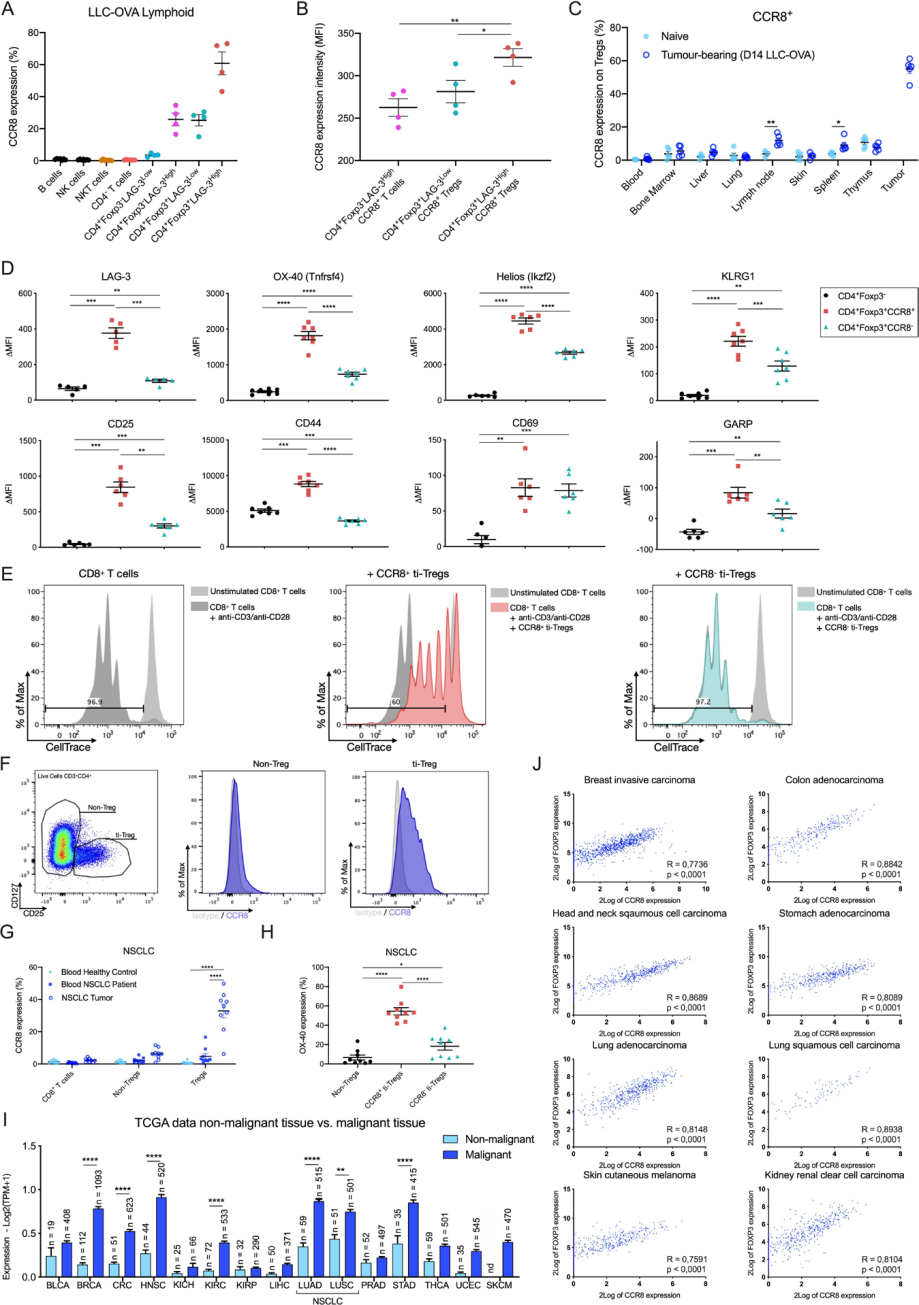
CCR8 is mainly expressed on the highly activated ti-Tregs. (A) Percentage CCR8^+^cells within different LLC-OVA tumor-infiltrating lymphoid cell subsets as measured via flow cytometry (n=5). (B) CCR8 expression level (MFI) on the CCR8^+^ tumor-infiltrating T-cell populations as measured via flow cytometry (n=4). (C) Percentage CCR8^+^cells within Tregs in different organs of tumor-bearing (dark blue) and naive C57BL/6 mice (light blue) as measured via flow cytometry (n=5). (D) Expression (ΔMFI) of LAG-3, OX-40, Helios, KLRG1, CD25, CD44, CD69 and GARP in the CD4^+^Foxp3^-^ (black circle), CD4^+^Foxp3^+^CCR8^+^ (red square) and CD4^+^Foxp3^+^CCR8^-^ (turquoise triangle) T-cell subsets as determined via flow cytometry (n=6). (E) Treg suppression assay. Splenic CD8^+^ T-cell proliferation after stimulation (anti-CD3+anti-CD28) in the presence of CCR8^+^ (red) or CCR8^−^ (turquoise) ti-Tregs at a ratio of one ti-Treg for five splenic T cells (1:5). (F) Representative flow cytometry plots showing Isotype/CCR8 expression (MFI) by non-Tregs and ti-Tregs of NSCLC patients. (G) Percentage CCR8^+^cells within different lymphoid subsets found in the blood or tumors of patients with NSCLC or healthy volunteers as measured via flow cytometry (n=9-11). (H) Percentage OX-40^+^cells within different lymphoid subsets found in the tumors of patients with NSCLC as measured via flow cytometry (n=9). (I) Expression of the *CCR8* gene within cancerous and surrounding healthy tissue of bladder urothelial carcinoma (BLCA), breast invasive carcinoma (BRCA), colorectal cancer (CRC), head and neck squamous cell carcinoma (HNSC), kidney chromophobe (KICH), kidney renal clear cell carcinoma (KIRC), kidney renal papillary cell carcinoma (KIRP), liver hepatocellular carcinoma (LIHC), lung adenocarcinoma (LUAD), lung squamous cell carcinoma (LUSC), prostate adenocarcinoma (PRAD), stomach adenocarcinoma (STAD), thyroid carcinoma (THCA), uterine corpus endometrial carcinoma (UCEC) and skin cutaneous melanoma (SKCM) (data retrieved from TCGA). (J) Correlation between the expression of *FOXP3* and *CCR8* in different human tumors (data retrieved from TCGA). (A to D and G to I) Data shown as mean±SEM. (B, D, G, H) *p<0.05, **p<0.01, ***p<0.001 and ****p<0.0001 by one-way ANOVA, (C, I) *p<0.05, **p<0.01 and ****p<0.0001 by (un)paired Student’s t-test. ANOVA, analysis of variance; LLC, Lewis Lung Carcinoma; NK, natural killer; NSCLC, non-small cell lung carcinoma; TCGA, The Cancer Genome Atlas; ti-Treg, tumor-infiltrating regulatory T cell.

We next evaluated the expression pattern of the CCR8 protein within tumor single cell suspensions via multicolor flow cytometry. No CCR8 could be detected at the surface of non-hematopoietic (CD45^−^) and myeloid (CD45^+^CD11b^+^) cell populations (gating strategy, online supplemental figure S2A). Within the lymphoid (CD45^+^CD11b^−^) subsets, CCR8 expression was observed on approximately 25% of the CD4^+^Foxp3^−^LAG-3^High^ cells (cluster CD4_S2) and CD4^+^Foxp3^+^LAG-3^Low^ Tregs (Treg_S2), and on approximately 60% of the CD4^+^Foxp3^+^LAG-3^High^ Tregs (Treg_S1) (figure 3A), but not on CD4-negative T cells (figure 3A, online supplemental figure S2A). These data largely corroborate the gene expression data, except for the CD8^+^LAG-3^High^ cells (CD8_S3), for which the observed presence of *Ccr8* messenger RNA does not translate at the protein level. Within the CCR8^+^ T cell fractions, CCR8 density (as measured by median fluorescence intensity (MFI)) appeared highest on the CD4^+^Foxp3^+^LAG-3^High^ Tregs (Treg_S1) (figure 3B). This CCR8 expression by ti-Tregs is not unique for the LLC-OVA model, with approximately 43% and 65% of all ti-Tregs staining positive for CCR8 in the MC38 and B16-OVA models, respectively (online supplemental figure S2B).

We next assessed CCR8 expression on Foxp3^+^ Tregs from distinct organs in LLC-OVA-bearing and naive mice (figure 3C). In naive animals, CCR8 was hardly detectable on Tregs from all tissues except the thymus, where approximately 8% of Tregs were CCR8^+^, illustrating that this is part of a normal physiological process in that organ (figure 3C). In tumor-bearing animals, CCR8^+^ Tregs were also observed in the thymus, but in addition also in the spleen (approximately 9% of the Tregs) and tumor-draining lymph node (approximately 12% of the Tregs) (figure 3C). However, this expression level was by far not as high as on Tregs in the TME, where CCR8 was found on approximately 57% of all Tregs (figure 3C). These data suggest a gradual increase in the percentage of CCR8^+^ Tregs from peripheral organs, to lymphoid organs, to the tumor site. Interestingly, also the density of CCR8 surface expression (as measured by MFI) on CCR8^+^ Tregs gradually increased from thymus, to spleen and further to tumor-draining lymph node and TME (online supplemental figure S2C).

We then assessed whether CCR8 is part of a larger Treg activation program, as suggested by the scRNA-seq data. CCR8^+^ ti-Tregs indeed showed a significantly higher surface expression level of Treg activation markers such as LAG-3, OX-40, Helios, KLRG1, CD25, CD44, CD69 and GARP in LLC-OVA (figure 3D), MC38 and B16-OVA tumors (online supplemental figure S2D). This correlated with a higher T-cell suppressive capacity of CCR8^+^ ti-Tregs compared with CCR8^-^ ti-Tregs, illustrated by a significant reduction of polyclonal CD8^+^ and CD4^+^ T-cell proliferation (figure 3E; online supplemental figure S2E). Collectively, these results indicate that CCR8 is predominantly expressed on highly activated and suppressive ti-Tregs.

We next assessed CCR8 expression on tumor-infiltrating T cells from freshly resected human NSCLC and melanoma tumor samples. CCR8 expression could be detected on approximately 40% of the CD3^+^CD4^+^CD127^−^CD25^+^ Tregs within NSCLC tumors (figure 3F, G). Interestingly, these CCR8^+^ ti-Tregs also showed significantly higher expression levels of OX-40, suggesting an overall higher activation state (figure 3H). Similar to the mouse model, a much smaller subset of CD4^+^CD127^+^CD25^−^ non-Treg cells were CCR8-positive, while CD8^+^ T cells were negative for CCR8 in these tumors (figure 3F, G). Notably, all these T-cell populations, including the Tregs, were mostly negative for CCR8 in the peripheral blood (figure 3G). Similar observations were made in patients with melanoma (online supplemental figure S2F), corroborating the notion that CCR8 expression can be used as a marker of ti-Tregs in distinct human cancer types. To further broaden this notion, we mined the TCGA platform to compare *CCR8* expression levels in distinct human tumors with their surrounding healthy tissue. *CCR8* expression was significantly upregulated in the TME of multiple tumor types (figure 3I), always showing a significant (p<0,0001) correlation with *FOXP3* expression (figure 3J). These data indicate FOXP3^+^ ti-Tregs as important *CCR8* expressors in human tumors.

### CCR8 is upregulated in response to TCR stimulation

**Figure 4:**
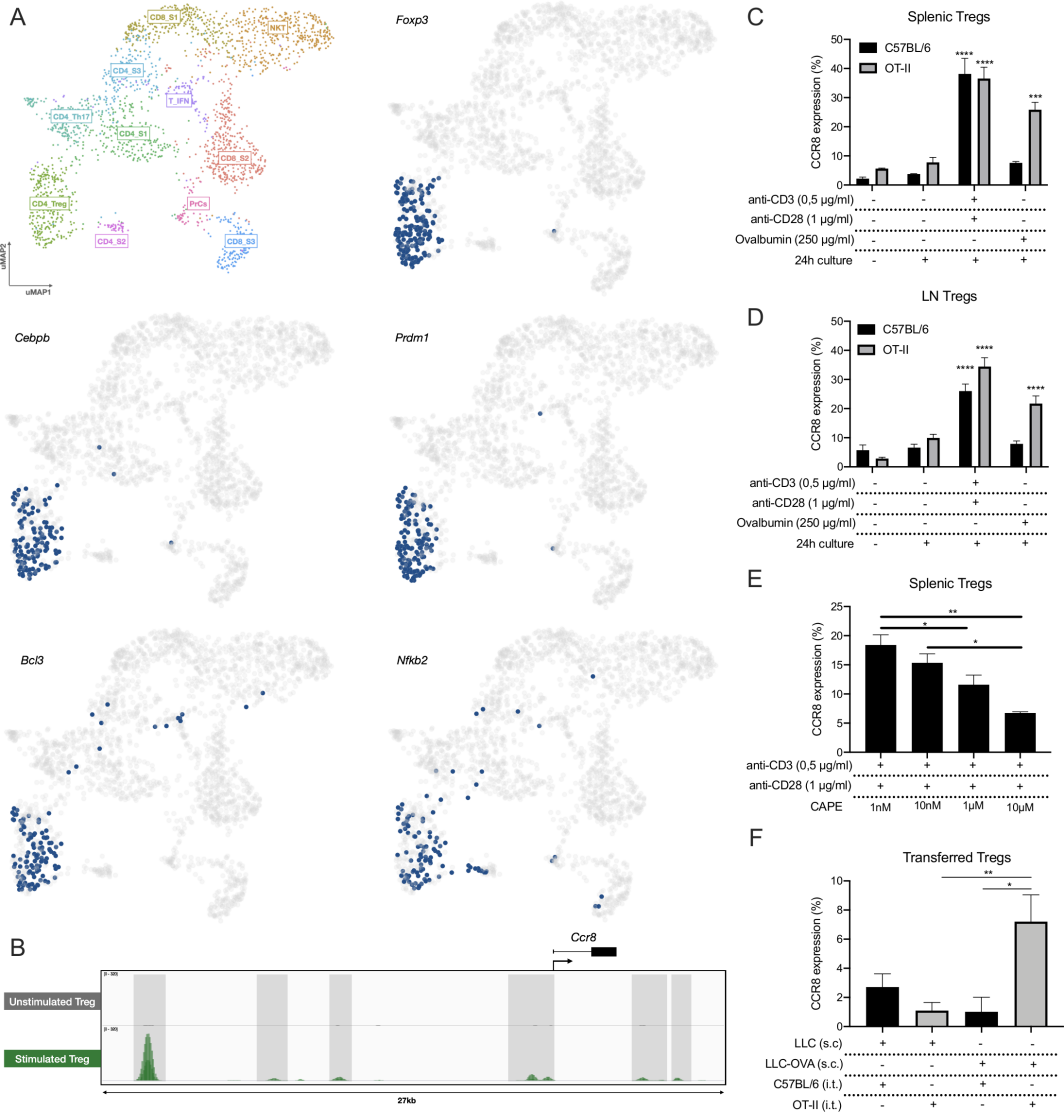
CCR8 upregulation is induced by TCR stimulation. (A) UMAP plots showing the activity of the *Foxp3*, *Cebpb*, *Prdm1*, *Bcl3* and *Nfkb2* regulons within the distinct T-cell subsets, cells in which the regulons are active are indicated in blue. (B) ChIP-seq (p65) signal profiles across the *Ccr8* locus. Data obtained from Oh *et al.*^42^ Peaks that are gained after Treg stimulation are highlighted (gray). (C) Percentage CCR8^+^ cells within C57BL/6 or OT-II splenic Tregs after 24 hours of in vitro co-culture with TCR stimulants (anti-CD3 +anti-CD28) or Chicken ovalbumin (OVA) (n=3). (D) Percentage CCR8^+^ cells within C57BL/6 or OT-II LN-derived Tregs after 24 hours of in vitro co-culture with TCR stimulants (anti-CD3+anti-CD28) or Chicken ovalbumin (OVA) (n=3). (E) Percentage CCR8^+^ cells within C57BL/6 splenic Tregs after 24 hours of in vitro co-culture with TCR stimulants (anti-CD3+anti-CD28) and distinct concentrations of CAPE (n=4). (F) Percentage CCR8^+^ cells within transferred C57BL/6 or OT-II splenic Tregs 48 hours after intratumoral (i.t.) adoptive transfer into LLC or LLC-OVA tumors (n=3). (C to F) Data shown as mean±SEM. (C, D) ***p<0.001 and ****p<0.0001 by one-way ANOVA where each condition was compared with prior to culture, (E, F) *p<0.05 and **p<0.01 by one-way ANOVA. ANOVA, analysis of variance; LLC, Lewis Lung Carcinoma; UMAP, Uniform Manifold Approximation and Projection.

We next aimed to understand the mechanism of CCR8 upregulation. First, we applied the SCENIC pipeline on the LLC-OVA tumor-infiltrating T/NKT cells to identify the master regulators that drive *Ccr8* expression in the distinct T-cell subsets.^41^ This allowed us to identify key regulons within each T-cell population, after which the cells were re-clustered based on their regulon activity and were colored by their matching Seurat clusters (online supplemental figure S3A). Within the CD4_Treg population, SCENIC identified several master regulators of the Treg phenotype, including *Foxp3, Cebpb*, *Prdm1*, *Bcl3* and *Nfkb2* (figure 4A, online supplemental figure S4). Of these, *Nfkb2* was predicted to be a *Ccr8* regulator and was shown to be active in the three *Ccr8* expressing T-cell clusters: CD4_Treg, CD8_S3 and CD4_S2 (figure 4A, online supplemental figure S4). Interestingly, NF-κB is strongly involved in TCR signaling, suggesting that T-cell activation could be a trigger for *Ccr8* expression. Analysis of genome-wide chromatin immunoprecipitation (ChIPseq) data of the NF-κB p65 subunit as published by Oh *et al*^42^ showed several p65 binding peaks associated with the *Ccr8* gene upon Treg stimulation (figure 4B). To validate these findings, we polyclonally stimulated total splenocytes, resulting in up to 40% of splenic Tregs becoming CCR8^+^ (figure 4C). Also CD4^+^Foxp3^−^ T cells turned CCR8^+^, although to a much lesser extent, whereas the CD8^+^ T cells showed no CCR8 upregulation (online supplemental figure S3B). To assess whether an antigen-specific T-cell activation yields the same effect, OT-II or control C57BL/6 splenocytes were cultured in the presence of ovalbumin. Only splenic OT-II Tregs significantly upregulated CCR8, demonstrating that antigen responsiveness is a determinant for CCR8 expression (figure 4C). The same results were obtained with Tregs derived from the lymph node (figure 4D, online supplemental figure S3C). Interestingly, splenocyte stimulation in the presence of the NF-κB inhibitor CAPE resulted in a dose-dependent reduction of CCR8 upregulation on the Tregs (figure 4E), whereas Treg viability was only slightly affected at the highest concentration (online supplemental figure S3D), highlighting the importance of NF-κB signaling for CCR8 regulation.

To verify whether antigen recognition also results in CCR8 upregulation in vivo, we adoptively transferred CellTrace-labeled OT-II splenic Tregs into the tumor of LLC-OVA-bearing mice, resulting in the upregulation of CCR8 expression on a fraction of these cells (figure 4F). No CCR8 upregulation was observed when the same OT-II Tregs were transferred into the tumors of LLC-bearing mice, indicating that a lack of antigen recognition prevents subsequent CCR8 induction. Together, these results demonstrate that CCR8 upregulation on ti-Tregs is induced in an NF-κB dependent manner and initiated by TCR stimulation through tumor antigen recognition.

### CCR8 is redundant for the recruitment, activation or suppressive capacity of ti-Tregs

**Figure 5:**
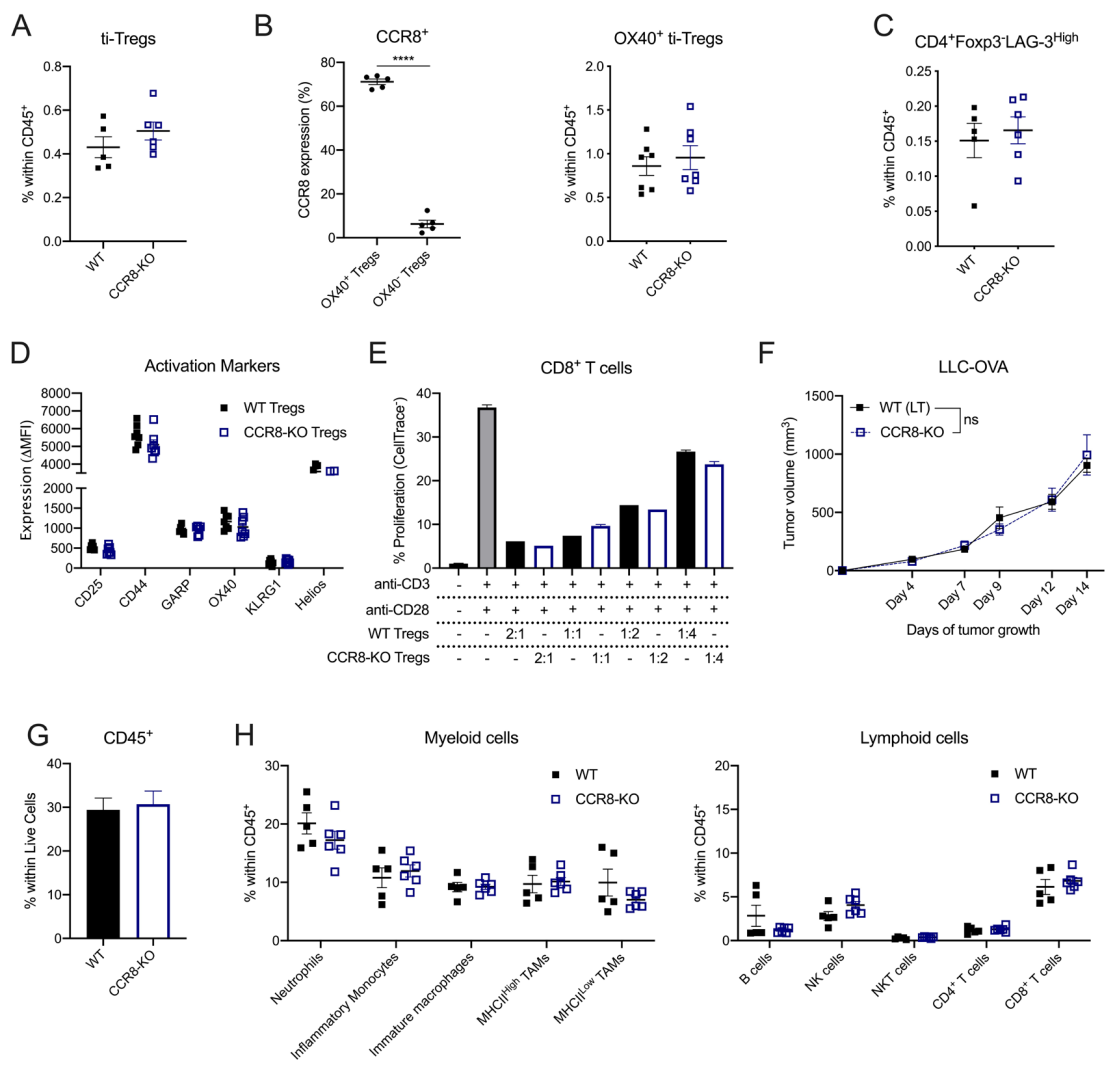
CCR8 is redundant for the functionality of ti-Tregs. (A) Percentage of Foxp3^+^ Tregs within the CD45^+^ population of LLC-OVA tumors in CCR8-KO (blue) and WT littermate (black) control mice (n=5–6). (B) Percentage of OX-40^+^ Tregs and OX-40^−^ Tregs that stains positive for CCR8 expression (n=5-6) (and) Percentage of OX-40^+^ Tregs within the CD45^+^ population of D14 LLC-OVA tumors in WT littermate (black) and CCR8-KO (blue) mice (n=7). (C) Percentage of CD4^+^Foxp3^−^LAG-3^high^ cells within the CD45^+^ population of LLC-OVA tumors in CCR8-KO (blue) and WT littermate (black) control mice (n=7). (D) Comparison of activation marker expression between ti-Tregs of the WT (black) and CCR8-KO (blue) TME in LLC-OVA tumors (n=7). (E) Treg suppression assay. Splenic CD8^+^ T-cell proliferation after stimulation (anti-CD3 + anti-CD28) in the presence of WT (black) or CCR8-KO (blue) ti-Tregs at distinct ti-Treg:splenic T cell ratios. (F) Tumor growth of s.c. injected LLC-OVA in CCR8-KO (blue) and WT littermate (black) control mice (n=7). (G) Influx of CD45^+^ cells within the TME of D13 LLC-OVA tumors grown in CCR8-KO (blue) or WT littermate (black) control mice (n=5-6). (H) Influx of distinct myeloid cell types (% of CD45^+^ cells) and lymphoid cell types (% of CD45^+^ cells) within the TME of D13 LLC-OVA tumors grown in CCR8-KO (blue) versus WT littermate (black) control mice (n=5–6). (A to H) Data shown as mean±SEM. (A to E, G, H) ****p<0.0001 by unpaired Student’s t-test, (F) by two-way ANOVA with Holm-Sidak multiple comparisons test. ANOVA, analysis of variance; LLC, Lewis LungCarcinoma; NK, natural killer; s.c., subcutaneously; TME, tumor microenvironment; ti-Treg, tumor-infiltrating regulatory T cell; WT, wild type.

To evaluate the potential roles of CCR8 on ti-Tregs, we first assessed whether CCR8 mediates Treg homing to the TME. The overall presence of ti-Tregs (figure 5A), as well as the percentage of activated CD4^+^Foxp3^+^OX-40^+^ ti-Tregs (Treg_S1), 70% of which are CCR8^+^, was unchanged within the CD45^+^ population of LLC-OVA tumors grown in CCR8-KO or WT littermate control mice (figure 5B). Also, the presence of CD4^+^Foxp3^-^LAG-3^High^ cells (CD4_S2), a fraction of which expresses CCR8 (figure 3A), was unaltered (figure 5C). These data argue against a dominant role for CCR8 in recruiting CCR8^+^ T cells towards the TME. Moreover, in LLC-OVA tumors, no significant differences in activation marker expression (figure 5D) nor T-cell suppressive capacity (figure 5E) could be observed between WT and CCR8-KO ti-Tregs, indicating that CCR8 is not majorly involved in Treg activation within the TME.

This lack of ti-Treg phenotype in CCR8-KO mice translated into an indistinguishable LLC-OVA tumor growth curve between CCR8-KO mice and WT littermate control mice (figure 5F), reflected by a similar immune composition of LLC-OVA tumors in both backgrounds (figure 5G, H, online supplemental figure S5, gating strategy online supplemental figure S6). Collectively, these results demonstrate that CCR8 is redundant for the recruitment, activation and suppressive capacity of ti-Tregs.

### Generation of tetravalent nanobody-Fc fusions that allow the specific blockade of CCR8 signaling

We next assessed CCR8 as a molecular target in therapeutic approaches that aim to mitigate the immunosuppressive ti-Treg population, without affecting Tregs in other tissues. Hereto, we generated anti-CCR8 nanobodies (Nbs, camelid single domain antibody fragments) through repeated immunization of llamas and alpacas with mouse CCR8-encoding plasmid DNA, as outlined in the online supplemental materials and methods. Phage display libraries derived from peripheral blood mononuclear cells were prepared and subjected to two consecutive selection rounds on HEK293T and CHO-K1 cells transiently transfected with mouse CCR8. Selected Nbs were then screened for binding to HEK293T cells transfected with either full length or N-terminally deleted mouse CCR8 (online supplemental table S1), in comparison to mock transfected cells, allowing their classification as N-terminal mouse CCR8 binders (only binding to full length CCR8) or extracellular loop mouse CCR8 binders (binding on full length and N-terminally deleted CCR8). Ultimately, two Nbs were chosen, with Nb-I requiring the presence of the N-terminus for binding, and Nb-II not (online supplemental figure S7A).

**Figure 6:**
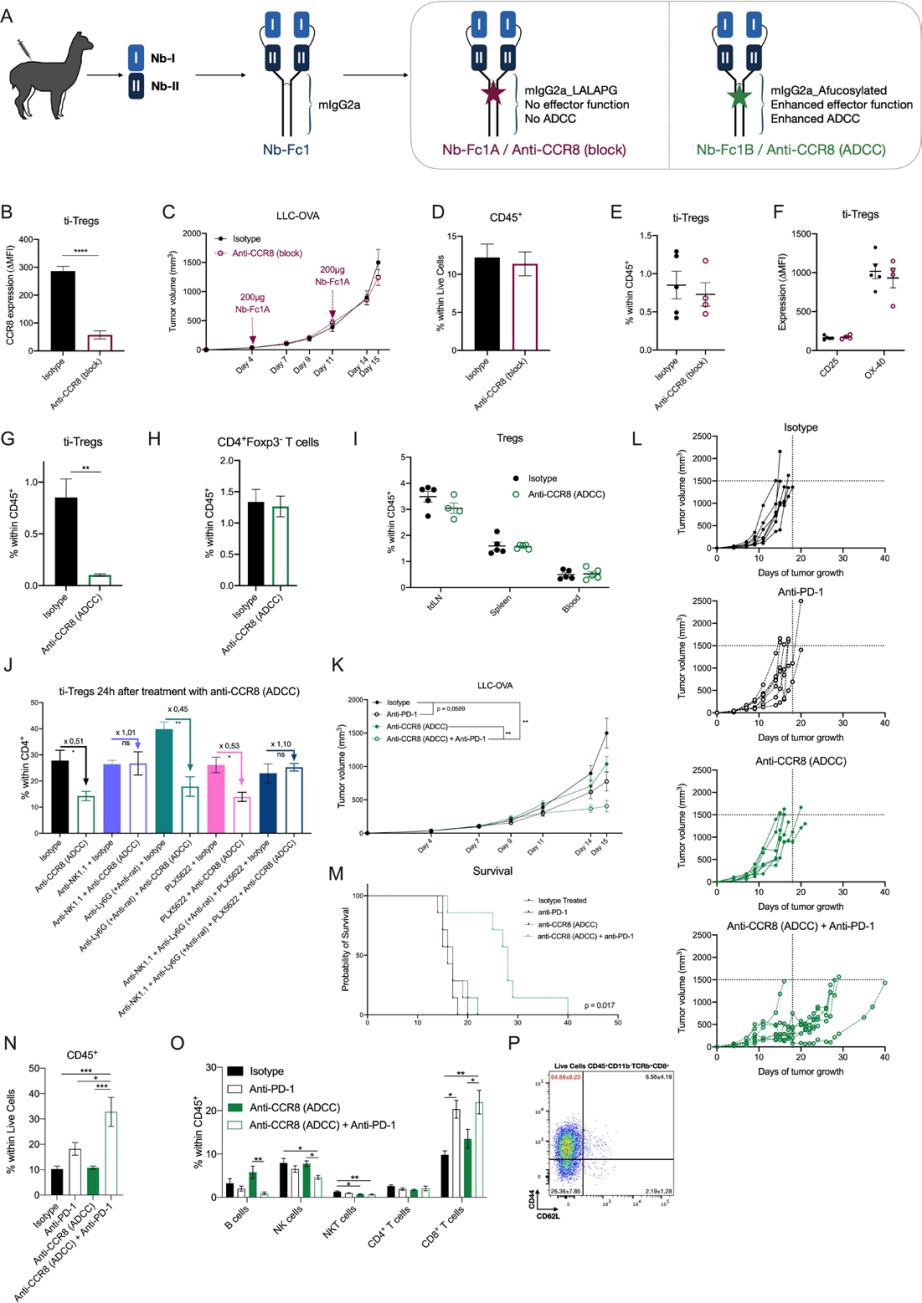
Specific depletion of CCR8^+^ ti-Tregs, but not CCR8 blockade, results in reduced LLC-OVA tumor growth and synergizes with anti-PD-1 therapy. (A) Schematic overview of tetravalent Nb-Fc generation. (B) Delta-MFI of CCR8 expression on ti-Tregs of isotype (black) or anti-CCR8 (block) (red) treated mice (n=5). (C) LLC-OVA tumor growth in isotype (black) or anti-CCR8 (block)-treated mice (red) (n=12). (D) Percentage of CD45^+^ hematopoietic cells within the TME of D16 LLC-OVA tumors of isotype (black) or anti-CCR8 (block)-treated mice (red) (n=5). (E) Percentage of CD4^+^Foxp3^+^ Tregs within the CD45^+^ compartment of D16 LLC-OVA tumors of isotype (black) or anti-CCR8 (block)-treated mice (red) (n=5). (F) Activation marker expression determined via flow cytometry, on ti-Tregs of D16 LLC-OVA tumors of isotype (black) or anti-CCR8 (block)-treated mice (red) (n=5). (G) Percentage of CD4^+^Foxp3^+^ Tregs within the CD45^+^ compartment of D16 LLC-OVA tumors of isotype (black) or anti-CCR8 (ADCC)-treated mice (green) (n=5). (H) Percentage of CD4^+^Foxp3^−^ T cells within the CD45^+^ compartment of D16 LLC-OVA tumors of isotype (black) or anti-CCR8 (ADCC)-treated mice (green) (n=5). (I) Percentage of CD4^+^Foxp3^+^ Tregs within the CD45^+^ compartment of distinct organs of isotype (black) or anti-CCR8 (ADCC)-treated (green) LLC-OVA tumor-bearing mice (n=5). (J) Percentage of CD4^+^Foxp3^+^ Tregs within the CD4^+^ compartment of D12 LLC-OVA tumors treated with isotype control or anti-CCR8 (ADCC) in combination with anti-NK1.1, anti-Ly6G (+ anti-rat) and/or PLX5622 (n=4). (K) s.c. LLC-OVA tumor growth in mice treated with isotype (black, closed circle), anti-PD-1 (black, open circle), anti-CCR8 (ADCC) (green, closed circle) or the combination of anti-PD-1 and anti-CCR8 (ADCC) (green, open circle) (n=12). (L) Growth of individual subcutaneous LLC-OVA tumors in mice treated with isotype (black, closed circle), anti-PD-1 (black, open circle), anti-CCR8 (ADCC) (green, closed circle) or the combination of anti-PD-1 and anti-CCR8 (ADCC) (green, open circle) (n=7). (M) Survival (tumor volume <1500 mm^3^) of LLC-OVA tumor-bearing mice treated with isotype (black, solid line), anti-PD-1 (black, dotted line), anti-CCR8 (ADCC) (green, solid line) or the combination of anti-PD-1 and anti-CCR8 (ADCC) (green, dotted line) (n=7). (N) Percentage of CD45^+^ hematopoietic cells within the TME of LLC-OVA tumors grown in mice treated with isotype, anti-PD-1, anti-CCR8 (ADCC) or the combination of anti-PD-1 and anti-CCR8 (ADCC) (n=5). (O) Percentage of distinct lymphocyte populations within the CD45^+^ compartment of LLC-OVA tumors grown in mice treated with isotype, anti-PD-1, anti-CCR8 (ADCC) or the combination of anti-PD-1 and anti-CCR8 (ADCC) (n=5). (P) Representative FACS plot showing the expression level of CD44 and CD62L on the CD8^+^ T cells within the TME of mice treated with a combination of anti-PD-1 and anti-CCR8 (ADCC) (n=5). (B to K, N to P) Data shown as mean±SEM. (B, D to J) *p<0.05, **p<0.01 and ****p<0.0001 by unpaired Student’s t-test, (C. K) **p<0.01 by two-way ANOVA with Holm-Sidak multiple comparisons test, (N, O) *p<0.05, **p<0.01 and ***p<0.001 by one-way ANOVA. ADCC, antibody-dependent cell-mediated cytotoxicity; anti-PD-1, anti-programmed cell death protein-1; ANOVA, analysis of variance; LLC, Lewis Lung Carcinoma; MFI, median fluorescence intensity; NK, natural killer; s.c., subcutaneously; ti-Treg, tumor-infiltrating regulatory T cell.

Nb-I and Nb-II were then combined into a tetravalent Nb-Fc fusion by covalent linking of Nbs with flexible peptide linkers (Nb-Fc1, figure 6A). Similar multivalent constructs are known to improve potency and efficacy through intermolecular and intramolecular avid binding on their respective chemokine receptor targets.^43–47^ Whereas Nb-Fc1 carries the wild type mouse IgG2a Fc moiety, Nb-Fc1A carries a LALAPG full-effector knockout version (no complement binding, fixation and antibody-dependent cell-mediated cytotoxicity (ADCC))^48^ and Nb-Fc1B carries an afucosylated Fc for enhanced ADCC function.^49^ The Nb-Fc fusions potently bind to mouse CCR8, with Nb-Fc1, Nb-Fc1A and Nb-Fc1B displaying comparable EC50 values in the nanomolar range of binding to endogenously expressed CCR8 on mouse BW5147 thymoma cells (online supplemental figure S7B). Next, dilution series of monovalent Nb-I and/or Nb-II were competed against Nb-Fc1 on BW5147 cells. An equimolar mixture of Nb-I and Nb-II appeared more efficient in displacing the binding of Nb-Fc1, compared with each of the nanobodies individually (online supplemental figure S7C), revealing that both nanobodies contribute to the avid binding of the Nb-Fc fusions. To assess their CCR8 blocking capacity, CCR8 expressing CHO-K1 cells were treated with the CCR8-ligand CCL1 and its inhibitory effect on cAMP accumulation was measured via homogenous time-resolved fluorescence detection (HTRF) in the presence or absence of Nb-Fc fusions. Nb-Fc1 potently blocked the action of CCL1 (online supplemental figure S7D). Similarly, the different Nb-Fc moieties, potently inhibited the protective effect of CCL1 against dexamethasone-induced apoptosis of BW5147 cells (online supplemental figure S7E). Together, these data support the notion that the Nb-Fc fusions potently inhibit the function of mouse CCR8 through the concomitant binding of Nb-I and Nb-II to distinct epitopes.

### Specific depletion of CCR8^+^ ti-Tregs, but not CCR8 blockade, results in reduced tumor growth and increased responsiveness to immune checkpoint blockade therapy

LLC-OVA tumor-bearing mice were treated with Nb-Fc1A, which blocks CCR8 but cannot perform any Fc-mediated function (termed Anti-CCR8 (block) in the Figures), and with Nb-Fc1B, which also blocks CCR8 but mediates ADCC (termed Anti-CCR8 (ADCC)). Intraperitoneal administration of 200 µg of Nb-Fc1A was done at days 4 and 11 of LLC-OVA tumor growth, resulting in an efficient occupation of CCR8 inside the TME (figure 6B), but also in the spleen (online supplemental figure S8A), as illustrated by the prevention of subsequent anti-CCR8 mAb binding. Nevertheless, no change in tumor growth was observed (figure 6C), along with an unaltered infiltration of CD45^+^ hematopoietic cells (figure 6D), various lymphoid cell populations (online supplemental figure S8B) and ti-Tregs in these tumors (figure 6E, gating strategy online supplemental figure S6). Moreover, the ti-Tregs expressed similar levels of activation markers (figure 6F). Notably, CCR8 blockade alone also did not affect the subcutaneous growth of MC38 colon carcinoma, a hot tumor that is known to be responsive to immunotherapy (online supplemental figure S8C). Overall, and in accordance with the observations in CCR8-KO mice, CCR8 blockade by itself appears not to be sufficient to affect tumor growth.

Treatment of LLC-OVA tumor-bearing mice with Nb-Fc1B (treatment schedule, online supplemental figure S9A) resulted in the complete depletion of CCR8^+^ Tregs in the TME (figure 6G), whereas CD4^+^Foxp3^−^ T cells remained unaffected (figure 6H). Importantly, Tregs in other organs, including the tumor-draining lymph node and spleen, remained unaffected (figure 6I), demonstrating the specificity of the treatment for ti-Tregs. Of note, a co-depletion of NK cells, but not macrophages or neutrophils (online supplemental figure S10A–C), prevented Nb-Fc1B-mediated Treg depletion (figure 6J), identifying NK cells as mediators of ADCC-dependent ti-Treg elimination.

The successful CCR8^+^ ti-Treg depletion resulted in a slightly reduced LLC-OVA tumor growth in comparison to isotype-treated mice (figure 6K, L). Of note, the minor antitumor effect of Nb-Fc1B was comparable to the effect of a monotherapy with anti-PD-1 in this model (figure 6K, L). Since both therapies aim to increase the antitumor T-cell response from different angles, we reasoned that their combination could have a beneficial effect. Interestingly, the combination of both therapies synergistically reduced tumor growth and significantly (p value=0.0171) prolonged survival (figure 6K–M), accompanied by a strong immune influx and increased levels of CD8^+^ T cells (figure 6N, O). Moreover, this CD8^+^ T-cell population consisted mostly of highly activated CD44^hi^ CD62L^lo^ effector cells (figure 6P). Importantly, no such synergism was observed in CCR8-KO mice (online supplemental figure S11A, B), demonstrating that the observed antitumor effects of Nb-Fc1B are indeed CCR8-specific.

Immune-related adverse events, such as colitis, can be a consequence of anti-PD-1 therapy.^50^ To assess for such events, anti-PD-1 and/or Nb-Fc1B treated mice were subjected to a thorough evaluation of gut inflammation by histological examination. No histological signs of gut inflammation were observed in colon and ileum sections of any of the treatment groups (online supplemental figure S9B), with no epithelial erosion, cell death, villus blunting, goblet cell loss nor elevated immune infiltration in any of the mice. Moreover, intestinal epithelial barrier permeability, as measured by lumen-to-blood passage of orally delivered 20 kDa FITC-dextran, did not increase after therapy (online supplemental figure S9C), nor did the mice show any weight loss (online supplemental figure S9D). Hence, while treatment with a combination of anti-PD-1 and anti-CCR8 (Nb-Fc1B) significantly affects tumor growth (figure 6K–M), it does not induce gut inflammation.

**Figure 7:**
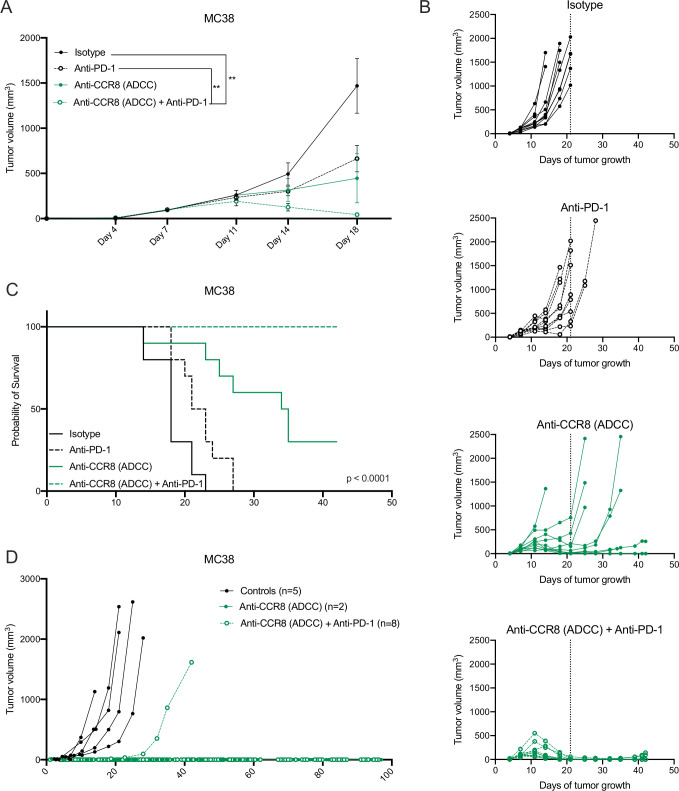
Specific depletion of CCR8^+^ ti-Tregs reduces MC38 tumor growth as monotherapy and synergizes with anti-PD-1 therapy. (A) s.c. MC38 tumor growth in mice treated with isotype (black, closed circle), anti-PD-1 (black, open circle), anti-CCR8 (ADCC) (green, closed circle) or the combination of anti-PD-1 and anti-CCR8 (ADCC) (green, open circle) (n=10). (B) Tumor growth of individual subcutaneous MC38 tumors grown in mice treated with isotype (black, closed circle), anti-PD-1 (black, open circle), anti-CCR8 (ADCC) (green, closed circle) or the combination of anti-PD-1 and anti-CCR8 (ADCC) (green, open circle) (n=10). (C) Survival (tumor volume <1500 mm^3^) of MC38 tumor-bearing mice treated with isotype (black, solid line), anti-PD-1 (black, dotted line), anti-CCR8 (ADCC) (green, solid line) or the combination of anti-PD-1 and anti-CCR8 (ADCC) (green, dotted line) (n=10). (D) Individual tumor volumes in mice subcutaneously re-challenged with MC38 after complete tumor regression on treatment with anti-CCR8 (ADCC) (green, closed circle) (n=2) or the combination of anti-PD-1 and anti-CCR8 (ADCC) (green, open circle) (n=8). (A) Data shown as mean±SEM. (A) **p<0.01 by two-way ANOVA with Holm-Sidak multiple comparisons test. ADCC, antibody-dependent cell-mediated cytotoxicity; anti-PD-1, anti-programmed cell death protein-1; ANOVA, analysis of variance; s.c., subcutaneously.

Strikingly, the antitumoral effects observed in the LLC-OVA model were even further enhanced in the MC38 model, where Nb-Fc1B monotherapy resulted in complete tumor rejection in 2 out of 10 mice and the combination of Nb-Fc1B and anti-PD-1 therapy resulted in complete tumor rejection in all mice after therapy ceased (figure 7A–C). Moreover, re-challenge of the complete responders with MC38 resulted in full tumor rejection in 2 out of 2 monotherapy and 7 out of 8 combination therapy-treated mice, indicative of a strong immunological memory (figure 7D). These results demonstrate that CCR8-targeted depletion of ti-Tregs unleashes antitumor immunity and synergizes with immune checkpoint blockade.

## Discussion

Recent studies in distinct human cancer types (breast, lung, colon and liver) have shown the unique upregulation of *CCR8* expression by ti-Tregs.^18–21^ Employing mouse tumor models, we demonstrated that *Ccr8* gene expression in the TME is indeed restricted to TCRβ^+^ cells. However, subsequent single-cell RNA sequencing of the LLC-OVA tumor-infiltrating TCRβ^+^ cell compartment revealed that *Ccr8* expression was not restricted to ti-Tregs, but could also be found in populations of dysfunctional CD4^+^ and CD8^+^ T cells. Hence, *Ccr8* is part of a common gene signature between Foxp3^+^ Tregs and Foxp3^−^ dysfunctional T cells, also including *Pdcd1, Lag3* and *Havcr2*. Remarkably, the CCR8 protein is differentially expressed by the *Ccr8*-positive cell types, with a high expression on ti-Tregs, a lower expression on dysfunctional CD4^+^ T cells and no detectable protein expression on dysfunctional CD8^+^ T cells. This appears to be an intrinsic difference between these T-cell types, since the polyclonal stimulation of C57BL/6 splenocytes or lymph node cells strongly induces CCR8 expression on Tregs and to a lesser extent on Foxp3^−^CD4^+^ T cells, but not on CD8^+^ T cells. Hence, high CCR8 expression is especially prominent on TCR-triggered, antigen-experienced Tregs. We demonstrated that the induction of CCR8 surface expression is at least partly dependent on NF-κB, which may initiate *Ccr8* gene expression by occupying *Ccr8* regulatory elements but may also have post-transcriptional effects. Alvisi *et al* hypothesized that *Ccr8* is part of a core network of immunosuppressive genes that is regulated by the transcription factor IRF4 in collaboration with BATF.^51^ These data could be reconciled by the finding that NF-κB and IRF4 may co-regulate the expression of genes associated with T-cell functions,^52^ possibly including *Ccr8*.

A more detailed analysis of the ti-Treg compartment at the single cell level revealed two distinct ti-Treg subsets, one of which showed a unique upregulation of *Ccr8* gene expression, co-expression of several activation markers and immune checkpoint molecules, and a superior suppressive activity. Similarly, protein expression of CCR8 in human NSCLC and melanoma tumors was restricted to the activated (OX-40^+^) CD3^+^CD4^+^CD127^−^CD25^+^ ti-Tregs. These findings are in line with the recent study on NSCLC patients where CCR8^+^ICOS^+^ ti-Tregs were identified as effector Tregs with a superior suppressive capacity.^51^

However, it remained unclear how specific CCR8 expression is for ti-Tregs, which is crucial information for the therapeutic applicability of CCR8-targeted approaches. In naive and tumor-bearing mice, CCR8 expression could be detected on a fraction of thymic Tregs. However, this thymic CCR8 expression was previously shown to be redundant for thymocyte development, with CCR8-KO mice showing normal thymocyte differentiation, motility and negative selection.^53^ Hence, CCR8-targeting is unlikely to interfere with normal T-cell development. Interestingly, in tumor-bearing mice, upregulation of CCR8 expression on Tregs could already be detected in the tumor-draining lymph node and spleen, but not in any other tissue except the thymus. These data may indicate Treg activation in the lymphoid organs prior to homing to the TME, as has been previously suggested.^54^ However, despite previous reports on a potential role for CCR8 in the recruitment,^54^ activation and/or suppressive capacity^55^ of Tregs, we observed that a lack of CCR8 expression or a Nb-mediated blockade of CCR8 signaling did not affect these Treg characteristics, nor did it affect tumor growth. This finding is in apparent contrast to Villarreal *et al*, where anti-CCR8 mAb therapy was reported to inhibit CCR8 signaling, resulting in slower tumor growth and improved survival in a mouse model of colon cancer.^56^ Since treatment with the mAb also resulted in a decreased frequency of CCR8^+^ ti-Tregs, it is likely that this anti-CCR8 mAb also resulted in Fc-mediated cell depletion.^56^

To solve the issue whether CCR8 blockade would be sufficient or whether the depletion of CCR8^+^ cells is required to enhance antitumor immunity, we generated strong CCR8-blocking tetravalent Nb-based moieties that only differ at their ADCC effector function, either ADCC-prone (afucosylated Fc) or ADCC-deficient (LALAPG mutated Fc). These unique novel tools clearly showed that (1) CCR8 blockade without ADCC is not sufficient for therapeutic efficacy and that (2) anti-CCR8 (ADCC-prone) Nb treatment resulted in the complete NK-mediated depletion of CCR8^+^ Tregs in the TME, without affecting Foxp3^−^CD4^+^ T cells or any Tregs in the periphery, strongly increasing the safety profile of this compound. This contrasts the treatment with the FDA (Food and Drug Administration)-approved CD25-blocking mAb daclizumab, which has been shown to mediate a prolonged depletion of CD4^+^CD25^+^ Tregs from the circulation^9^ and the anti-mouse CD25 mAb (PC61) depleting CD4^+^CD25^+^ Tregs from peripheral lymphoid tissues.^12^

Our data show an antitumor effect of the anti-CCR8 (ADCC-prone) monotherapy, but especially a synergistic action with anti-PD-1. It has been shown that anti-PD-1 treatment may lead to the expansion of ti-Tregs and hyperprogression of the tumors,^57 58^ a phenomenon which would be counteracted by the concomitant depletion of ti-Tregs. The reduction in tumor growth of the combination therapy could be attributed to a more immunogenic TME rich in effector CD8^+^ T cells. This altered balance between suppressive ti-Tregs and effector CD8^+^ T cells has been reported to be crucial for an effective antitumor immune response.^5 59^

It has previously been shown that a fraction of patients treated with anti-PD-1 are prone to develop immune-related adverse events (irAEs) such as colitis and particularly when anti-PD-1 is combined with other immune checkpoint inhibitors such as anti-CTLA-4.^50^ However, no signs of irAEs including gut inflammation were observed in any of our treatment groups.

Together our study compiles strong evidence to suggest that a specific depletion of ti-Tregs, using the ti-Treg specific marker CCR8 as molecular target, could be used as a powerful additive in current immunotherapies. The finding that CCR8 upregulation can be detected on effector ti-Tregs of multiple human cancer types by both this study and previous studies^18–23^ supports a broad applicability of CCR8^+^ cell-depleting therapeutics in the clinic.
