# Supplementary material for: Therapeutic depletion of CCR8+ tumor-infiltrating regulatory T cells elicits antitumor immunity and synergizes with anti-PD-1 therapy
Source: J Immunother Cancer. 2021 Feb 15;9(2):e001749. doi: 10.1136/jitc-2020-001749 (PMC7887378; doi:10.1136/jitc-2020-001749)
Supplement: Supplementary data [file jitc-2020-001749supp001.pdf]

## SUPPLEMENTARY DATA

Supplementary Materials and Methods

Supplementary References

Figure S1. Additional data on single cell RNA-seq analysis of the LLC-OVA tumor-infiltrating T-cell compartment.

Figure S2. Additional data on CCR8$^+$ ti-Tregs in distinct cancer models.

Figure S3. Additional data on CCR8 upregulation in response to TCR stimulation.

Figure S4. Regulon activity matrix as determined using SCENIC.

Figure S5. CD8$^+$ T cell activation status in CCR8-KO mice.

Figure S6. Myeloid and Lymphoid gating strategies.

Figure S7. Generation of tetravalent Nb-Fc fusions that allow specific blockade of CCR8 signaling.

Figure S8. Additional data on CCR8 blockade.

Figure S9. Therapeutic treatment of LLC-OVA tumor-bearing mice.

Figure S10. In vivo cell depletion.

Figure S11. Anti-CCR8 (ADCC) therapy does not have a therapeutic effect in CCR8-KO mice.

Table S1. Amino acid sequences of the CCR8 fragments used for immunization/transfection.

Table S2. Clinical characteristics of NSCLC and melanoma patients that participated in this study.

Table S3. Antibodies used in the flow cytometric analysis of both mouse and human samples.

## Supplementary Materials and Methods

*Ex vivo single-cell preparation*

Tumor single cell suspensions were obtained by cutting the respective tissues in small pieces, followed by treatment with 10 U $ml^{-1}$ collagenase I, 400 U $ml^{-1}$ collagenase IV and 30 U $ml^{-1}$ DNaseI (Worthington) for 25 minutes at 37°C. The tumors were subsequently squashed and filtered (70µm). Spleens were squashed and filtered (70µm). The obtained cell suspensions were removed of red blood cells using erythrocyte lysis buffer (155mM NH4Cl, 10mM KHCO3, 500mM EDTA), followed by neutralization with RPMI, in 3-5 consecutive rounds. Tumor-draining lymph node (LN), Bone marrow *[1]*, thymus *[2]*, liver *[3]*, intestines *[4]*, and skin *[5]* single cell suspensions were prepared as previously described.

*Flow cytometry and cell sorting of mouse/human samples*

After erythrocyte lysis, the obtained single cell suspensions were resuspended in FACS buffer (PBS enriched with 2% FCS and 2mM EDTA) and counted. The antibodies that were used for cell surface staining and intracellular staining are listed in Supplementary table S3. All single cell suspensions were pre-incubated with rat anti-mouse CD16/CD32 (2.4G2; BD Biosciences) or anti-human Fc block reagent (Miltenyi) for 15 minutes prior to staining. After washing, the samples were stained with fixable viability dye eFluor506 (eBioscience) (1:200) for 30 minutes at 4°C and in the dark. Subsequently, the samples were washed and stained for 30 minutes at 4°C in the dark. The intracellular staining of cytokines/chemokines and transcription factors was done according to the manufacturer's protocol (Cat N° 554715; BD Biosciences) and (Cat N° 00-5523; Invitrogen), respectively. Delta median fluorescence intensity (ΔMFI) was determined as: (MFI staining) – (MFI isotype control). FACS data were acquired using the BD FACSCantoII (BD Biosciences) and analyzed using FlowJo (TreeStar, Inc.). To purify T-cell populations from the spleen, $CD90.2^{+}$ cells were MACS-enriched (anti-CD90.2 microbeads; Miltenyi). To purify Treg populations from the spleen, $CD4^{+}CD25^{+}$ cells were MACS-enriched ($CD4^{+}CD25^{+}$ Regulatory T cell isolation kit, mouse; Miltenyi Biotec). To purify $Tregs^{Thy1.1}$ from the tumor; $Thy1.1^{+}$ Tregs were MACS-enriched (anti-CD90.1 microbeads; Miltenyi) and subsequently sorted ($CD45^{+}CD11b^{-}TCR\beta^{+}CD4^{+}Thy1.1^{+}$) using a BD FACSAria II (BD Biosciences) from 5-7 pooled tumors. To purify the $CD45^{+}$ and the $CD45^{-}$ fraction from D14 LLC-OVA tumors, they were sorted from three separate tumors using a BD FACSAria II (BD Biosciences). To purify the $CD45^{+}CD11b^{+}$ and the $CD45^{+}CD11b^{-}TCR\beta^{+/-}$ fraction from D14 LLC-OVA tumors, the $CD45^{+}$ cells were MACS enriched (anti-CD45 microbeads; Miltenyi) and the subsets were subsequently sorted from three separate tumors using a BD FACSAria II (BD Biosciences).

*RNA extraction and cDNA preparation for qPCR*

RNA was extracted from whole tumor single cell suspensions (Day 14 LLC-OVA) or the sorted fractions using TRIzol (Invitrogen). The obtained RNA samples were reverse transcribed using oligo(dT) primers and SuperScript II RT (Invitrogen). Subsequently, quantitative real-time PCR was performed using an iCycler and iQ SYBR Green Supermix (Bio-Rad). PCR cycles consisted of 1 minute at 94°C, 45 seconds at 55° and 1 minute at 72°C. *Ccr8* expression was determined using the 5'-GGGACTGCGATGTGTAAGGT-3' forward and 5'-

TTGTAGCATGCCGTCTTCAG-3’ reverse primers and was normalized to the *S12* housekeeping gene.

*Suppression assays and OT-II T cell activation*

For the Treg suppression assay, $10^4$ $CD90.2^+$ splenocytes were labeled with CellTrace Violet (Thermo Fister Scientific) and co-cultured with distinct ratios of unlabeled $CD45^+CD11b^-$ $TCR\beta^+CD4^+Thy1.1^+$ $CCR8^+$, $CCR8^-$, WT or CCR8-KO ti-Tregs. T-cell proliferation was stimulated through the addition of 0.5 µg $ml^{-1}$ of soluble anti-CD3 (clone 145-2C11) (generated in house) and 1 µg $ml^{-1}$ of anti-CD28 (clone 37.51) (eBioscience) antibodies. The cells were cultured in round-bottom 96-well plates for three days. Proliferation of the CellTrace Violet labeled cells was determined via flow cytometry. For the stimulation of splenic T cells from C57BL/6 or OT-II mice, the splenic single cell suspension was stimulated through the addition of 1 µg $ml^{-1}$ of soluble anti-CD3 and 2 µg $ml^{-1}$ of anti-CD28 antibodies. Alternatively, cells were stimulated by adding Chicken Ovalbumin (250µg $ml^{-1}$) (Sigma). The cells were cultured in a flat-bottom 96-well plate for 24 hours.

*Immunizations and phage display selections*

Animals were immunized four times at two-week intervals with 2mg of mouse CCR8-encoding DNA (Supplementary Table S3) in pVAX1 (ThermoFisher Scientific, catalogue # V26020), after which blood samples were taken. Three months later, all animals were re-immunized with 2 mg of the same DNA, after which blood samples were taken. Phage display libraries derived from peripheral blood mononuclear cells (PBMCs) were prepared and used as previously described *[6,7]*. The Nb fragments were cloned into a M13 phagemid vector containing MYC and His6 tags. The library was rescued by infecting exponentially growing *Escherichia coli* TG1 [(F′ traD36 proAB lacIqZ ΔM15) supE thi-1 Δ(lac-proAB) Δ(mcrB-hsdSM)5(rK− mK−)] cells followed by superinfection with the VCSM13 helper phage. The mouse CCR8 DNA immunized phage libraries were subjected to two consecutive selection rounds on HEK293T cells transiently transfected with mouse CCR8 in pVAX1 followed by CHO-K1 cells transiently transfected with mouse CCR8 in pVAX1. Polyclonal phagemid DNA was prepared from *E. coli* TG1 cells that were infected with the eluted phages from the second rounds. The Nb fragments were amplified by means of PCR from these samples and subcloned into an *E. coli* expression vector, in frame with N-terminal PelB signal peptide and C-terminal FLAG3 and His6 tags. Electrocompetent *E. coli* TG1 cells were transformed with the resulting Nb-expression plasmid ligation mixture and individual colonies were grown in 96-deep-well plates. Monoclonal Nbs were expressed essentially as described before *[6]*. The crude periplasmic extracts containing the Nbs were prepared by freezing the bacterial pellets overnight followed resuspension in PBS and centrifugation to remove cell debris.

*Screening of CCR8 selection outputs*

Comparison of the binding (median fluorescent intensity) signal of a given Nb clone across the three cell lines enabled its classification as an N-terminal mouse CCR8 binder (binding on mouse CCR8 cells, but not on mouse CCR8 (delta16-3XHA) or control cells) or an extracellular loop mouse CCR8 binder (binding on mouse CCR8 cells and mouse CCR8 (delta16-3XHA), but not on control cells). Hundreds of clones were screened this way followed by sequence

determination of hits. Synthetic DNA fragments encoding a number of CCR8 binding Nbs were ordered and subcloned into an *E. coli* expression vector under control of an IPTG-inducible lac promoter, in frame with N-terminal PelB signal peptide (which directs the recombinant proteins to the periplasmic compartment) and C-terminal FLAG3 and His6 tags. Electrocompetent *E. coli* TG1 cells were transformed and the resulting clones were sequence verified. Nb proteins were purified from these clones by means of IMAC chromatography followed by desalting according to well established procedures *[6]*.

*Cloning, expression and purification of Nb-Fc fusions*

Tetravalent Nb-Fc constructs were generated combining a copy each of Nb-I and Nb-II per Fc arm separated by a 10 amino acid flexible GlySer linker and fused by a second 10 amino acid flexible GlySer linker to the hinge region of the mouse IgG2a Fc domain. These were cloned in a pcDNA3.4 mammalian expression vector, in frame with the mouse Ig heavy chain V region 102 signal peptide to direct the expressed recombinant proteins to the extracellular environment. DNA synthesis and cloning, cell transfection, protein production in Expi293F cells and protein A purification were done by Genscript (GenScript Biotech B.V., Leiden, Netherlands). Alternatively, constructs were cloned in mammalian expression vector pQMCF in frame with a secretory signal peptide and transfected to CHOEBNALT85 1E9 cells, followed by expression, protein A and gel filtration chromatography (Icosagen Cell Factory, Tartu, Estonia). Nb-Fc fusions with afucosylated N-glycans in the CH2 domain of the Fc moiety were obtained from expressions in a CHOEBNALT85 cell line that carries GlymaxX technology (ProBioGen AG, Berlin, Germany) (Icosagen Cell Factory, Tartu, Estonia).

*Binding and competition experiments*

HEK293T cells transiently transfected with mouse CCR8 in pVAX1 were recovered using cell dissociation non-enzymatic solution (Sigma Aldrich, catalogue # C5914-100ML) and resuspended to a final concentration of 1.0 x $10^6$ cells/ml in FACS buffer. Dilutions (1:5 in FACS buffer) of crude periplasmic extracts containing Nbs were incubated with mouse anti-FLAG biotinylated antibody (Sigma Aldrich, catalogue # F9291-1MG) at 5 µg/ml in FACS buffer for 30 min at RT with shaking. Cell suspensions were distributed into a 96-well v-bottom plate and incubated with the above described Nb/antibody mixture for 1 hour on ice while shaking. Nb binding to cells was detected with streptavidin R-PE (Invitrogen, catalogue # .SA10044) at 1:400 dilution (0.18 µg/ml) in FACS buffer, incubated for 30 minutes on ice with shaking and protection from light. Surface expression of mouse CCR8 on transiently transfected cell lines was confirmed by means of PE anti-mouse CCR8 (Biolegend, catalogue # 150311) antibody at 2 µg/ml. Nb-Fc fusions were evaluated for their binding to mouse CCR8 endogenously expressed on BW5147 cells [8–11] by means of flow cytometry experiments. Cells were incubated with different concentrations of Nb-Fc fusions for 30 minutes at 4°C, followed by two washes with FACS buffer, followed by 30 minutes incubation at 4°C with AF488 goat anti mouse IgG (Life technologies, catalogue # A11029), followed by two washing steps. Dead cells were stained using TOPRO3 (Thermo Fisher Scientific, catalogue # T3605). For binding competition experiments, cells were first incubated with different concentrations of monovalent Nb building blocks for 30 minutes at 4°C, followed by a 30 minutes incubation at 4°C of a fixed concentration of Nb-Fc fusions, followed by washing and detection of the Fc-moiety as described above.

*cAMP HTRF assay for Gi-coupled receptors*

CHO-K1 cells stably expressing recombinant mouse CCR8 receptor were grown prior to the test in media without antibiotic and detached by gentle flushing with PBS-EDTA (5 mM EDTA), recovered by centrifugation and resuspended in assay buffer (KRH: 5 mM KCl, 1.25 mM MgSO4, 124 mM NaCl, 25 mM HEPES, 13.3 mM Glucose, 1.25 mM KH2PO4, 1.45 mM CaCl2, 0.5 g/l BSA, supplemented with 1mM IBMX). 12 µl of cells were mixed with 6 µl of anti-CCR8 Nbs at ten concentrations in duplicate and then incubated for 30 min. Thereafter, 6 µl of a mixture of forskolin (to a final concentration of 5 µM) and mouse CCL1 (R&D Systems, catalogue # 845-TC) was added (to a final concentration of 1 nM corresponding to its EC80 value). The plates were then incubated for 30 min at room temperature. After addition of the lysis buffer and 1hour incubation, fluorescence ratios were measured according to the manufacturer's specification, with the HTRF kit (Cisbio, catalogue # 62AM9PE).

*Apoptosis assay*

Dexamethasone induces cell death in mouse lymphoma BW5147 cells that endogenously express CCR8. This cell death can be reversed by addition of CCL1, an agonistic ligand of CCR8 [8–11]. A dexamethasone-sensitive clone of BW5147 was kindly provided by Jean-Christophe Renauld and Laure Dumoutier (UCLouvain). 50 µl of cells (seeded at 2.75 x 104 cells/ml in Iscove-Dulbecco's medium + 10% FCS, 50 µM 2-ME, 1,25 mM l-glutamine; no Phenol red) were incubated with 30 µl of serial dilutions of anti-CCR8 Nbs and incubated for 30 minutes at 37°C. Next, a 20 µl mixture of dexamethasone (Sigma-Aldrich, catalogue # D4902) and human CCL1 (Biolegend, catalogue # 582706) was added to a final concentration of 10 nM each. After 48 hours incubation at 37°C, cell viability was quantified using the ATPlite 1 step kit according to the manufacturer's instructions (Perkin Elmer, catalogue # 6016736).

*Histology*

Mouse intestinal tissues (colon and ileum) were fixed in formalin (10%; neutral buffered; Sigma– Aldrich). Samples were dehydrated, embedded in paraffin, sectioned at 4 µm, stained with hematoxylin and eosin (H&E). For combined Alcian Blue and periodic acid Schiff stainings, dewaxed sections were rehydrated and incubated in Alcian Blue for 20 min. Sections were subsequently washed with PBS before incubation in 1% periodic acid for 10 min followed by incubation in Schiff 's reagent for 10 min. Sections were counterstained with Mayer's hematoxylin for 30 sec, washed and dehydrated before mounting with Depex. All slides were scanned with a Zeiss slide scanner.

*FITC–dextran intestinal permeability assay*

Intestinal permeability was assessed by oral gavage of FITC-labeled dextran (Sigma–Aldrich). Mice were administered 600 mg FITC-labeled dextran per kg body weight in sterile phosphate-buffered saline (PBS) by oral gavage. After 4 hours, mice are euthanised blood was collected from the facial vein, and FITC-labeled dextran levels were measured in 100 µl serum by fluorometry (485 nm). Mice were anesthetized during blood collection. Serial dilutions of FITC-

labeled dextrane in PBS were used each time to generate a standard curve, and serum from PBS-gavaged mice was used as blanks.

**Supplementary references**

1 Laoui D, Keirsse J, Morias Y, *et al.* The tumour microenvironment harbours ontogenically distinct dendritic cell populations with opposing effects on tumour immunity. *Nat Commun* 2016;**7**:13720. doi:10.1038/ncomms13720

2 Thyagarajan HM, Lancaster JN, Lira SA, *et al.* CCR8 is expressed by post-positive selection CD4-lineage thymocytes but is dispensable for central tolerance induction. *PLoS ONE* 2018;**13**:e0200765. doi:10.1371/journal.pone.0200765

3 Scott CL, Zheng F, De Baetselier P, *et al.* Bone marrow-derived monocytes give rise to self-renewing and fully differentiated Kupffer cells. *Nat Commun* 2016;**7**:10321. doi:10.1038/ncomms10321

4 Bain CC, Mowat AMcI. CD200 receptor and macrophage function in the intestine. *Immunobiology* 2012;**217**:643–51. doi:10.1016/j.imbio.2011.11.004

5 McCully ML, Collins PJ, Hughes TR, *et al.* Skin Metabolites Define a New Paradigm in the Localization of Skin Tropic Memory T Cells. *JI* 2015;**195**:96–104. doi:10.4049/jimmunol.1402961

6 Pardon E, Laeremans T, Triest S, *et al.* A general protocol for the generation of Nanobodies for structural biology. *Nat Protoc* 2014;**9**:674–93. doi:10.1038/nprot.2014.039

7 Henry KA, MacKenzie CR. Editorial: Single-Domain Antibodies—Biology, Engineering and Emerging Applications. *Front Immunol* 2018;**9**:41. doi:10.3389/fimmu.2018.00041

8 Denis C, Deiteren K, Mortier A, *et al.* C-Terminal Clipping of Chemokine CCL1/I-309 Enhances CCR8-Mediated Intracellular Calcium Release and Anti-Apoptotic Activity. *PLOS ONE* 2012;**7**:e34199. doi:10.1371/journal.pone.0034199

9 Louahed J, Struyf S, Demoulin J-B, *et al.* CCR8-dependent activation of the RAS/MAPK pathway mediates anti-apoptotic activity of I-309/CCL1 and vMIP-I. *European Journal of Immunology* 2003;**33**:494–501. doi:10.1002/immu.200310025

10 Spinetti G. The chemokine receptor CCR8 mediates rescue from dexamethasone-induced apoptosis via an ERK-dependent pathway. *Journal of Leukocyte Biology* 2003;**73**:201–7. doi:10.1189/jlb.0302105

11 Van Snick J, Houssiau F, Proost P, *et al.* I-309/T cell activation gene-3 chemokine protects murine T cell lymphomas against dexamethasone-induced apoptosis. *Journal of immunology (Baltimore, Md : 1950)* 1996;**157**:2570–6.

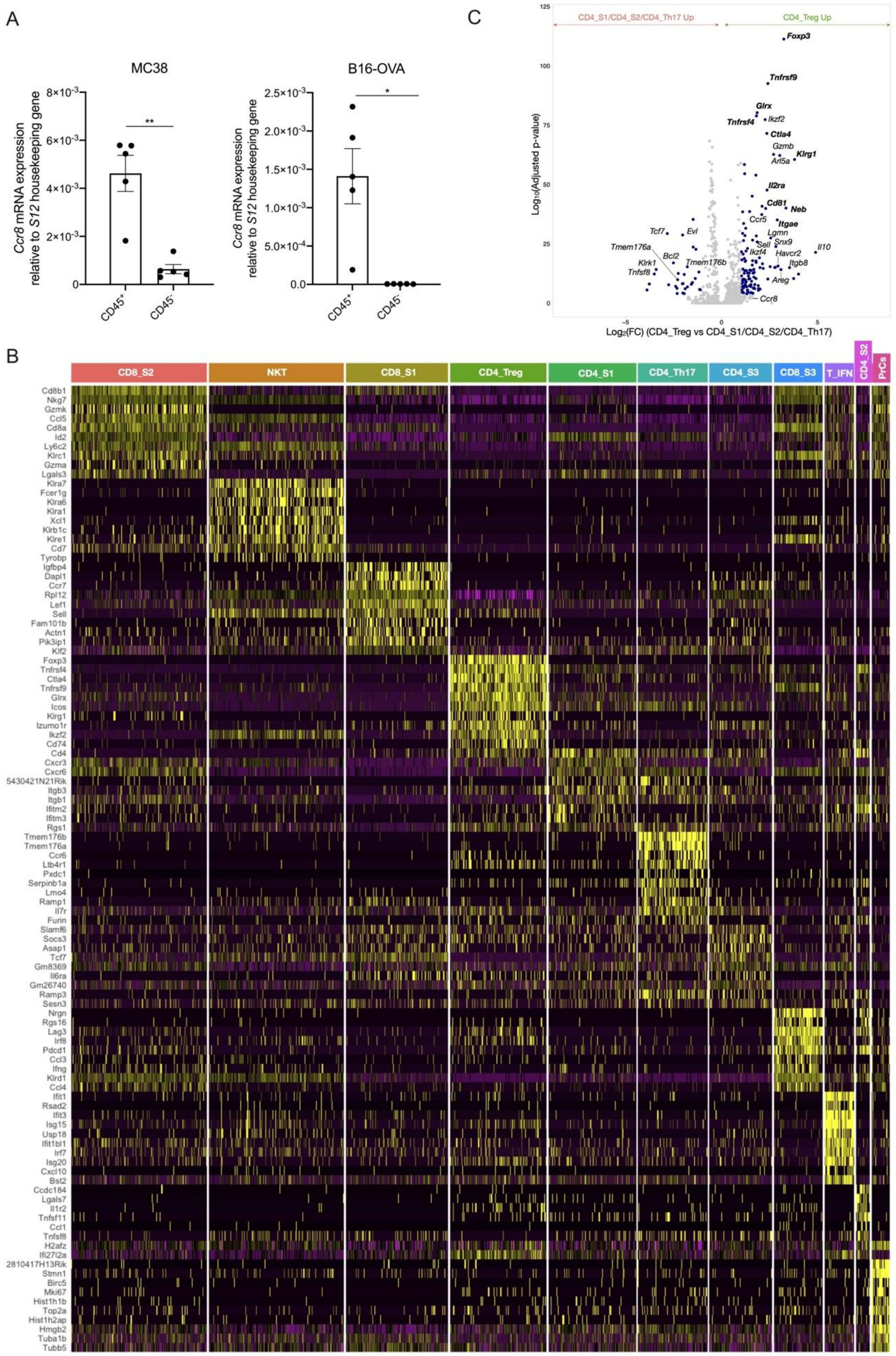
A
MC38
B16-OVA
Ccr8 mRNA expression relative to S12 housekeeping gene
CD45+
CD45-
C
CD4_S1/CD4_S2/CD4_Th17 Up
CD4_Treg Up
Log10(Adjusted p-value)
Log2(FC) (CD4_Treg vs CD4_S1/CD4_S2/CD4_Th17)
Foxp3
Tnfrsf9
Glrx
Tnfrsf4
Ikzf2
Ctla4
Klrg1
Il2ra
Cd81
Neb
Itgae
Il10
Ccr8
B
CD8_S2
NKT
CD8_S1
CD4_Treg
CD4_S1
CD4_Th17
CD4_S3
CD8_S3
T_IFN
CD4_S2
PrCs

**Figure S1:** Additional data on single cell RNA-seq analysis of the LLC-OVA tumor-infiltrating T-cell compartment. **A.** *Ccr8* expression in the $CD45^+$ hematopoietic cell subset and $CD45^-$ non-hematopoietic cell subset of MC38 and B16-OVA tumors as measured via qRT-PCR (n=5). **B.** Heatmap showing the 10 most upregulated genes in each individual (NK)T-cell subset from LLC-OVA tumors. **C.** Volcano plot showing the genes that are differentially expressed between CD4_Treg and all other $CD4^+$ T-cell subsets. **(A-B)** Data shown as mean±SEM. **(A, B)** * $p<0.05$ and ** $p<0.01$ by paired Student's t-test.

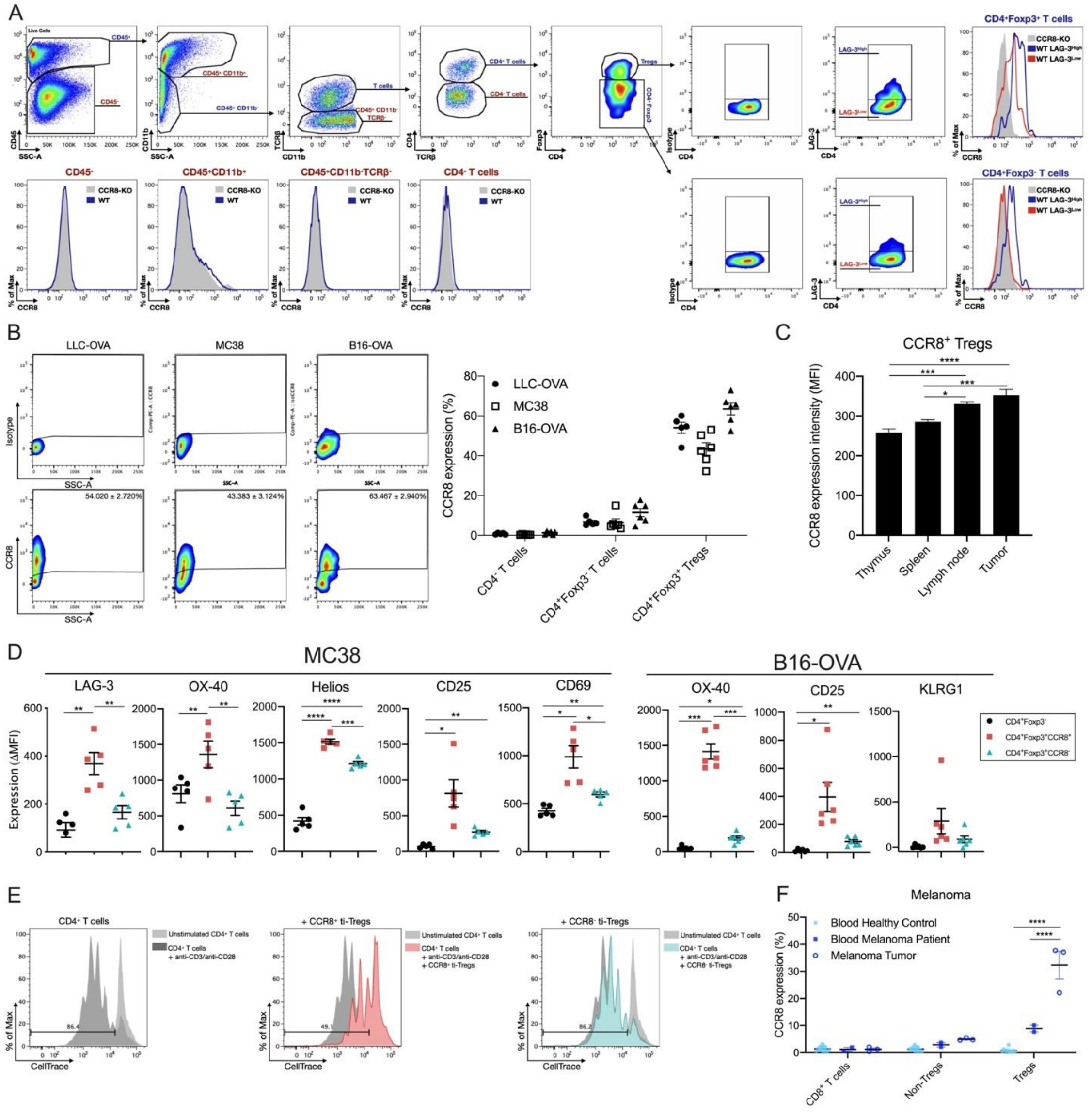


**Figure S2:** Additional data on CCR8+ ti-Tregs in distinct cancer models. **A.** LAG-3High and LAG-3Low ti-Treg gating strategy with CCR8 expression in each indicated cell type. **B.** *Left* Representative FACS plot showing the expression level of CCR8 on the ti-Tregs of LLC-OVA, MC38 and B16-OVA tumors. *Right* CCR8 expression by different LLC-OVA, MC38 and B16-OVA tumor-infiltrating lymphoid cell subsets measured via flow cytometry (n=5-6). **C.** CCR8 expression level on the CCR8+ Tregs found in the thymus, spleen, tumor-draining lymph node and tumor of LLC-OVA tumor-bearing mice (n=5). **D.** Expression level of several activation markers in the CD4+Foxp3-, CD4+Foxp3+CCR8+ and CD4+Foxp3+CCR8- T-cell subsets of MC38 and B16-OVA tumors as determined by flow cytometry (n=5-6). **E.** Treg suppression assay. Splenic CD4+ T-cell proliferation after stimulation (anti-CD3 + anti-CD28) in the presence of CCR8+ or CCR8- ti-Tregs at a ratio of 1 ti-Treg for 5 splenic T cells (1:5). **F.** Percentage CCR8+ cells within different lymphoid subsets found in the blood or tumors of melanoma patients or healthy volunteers as measured via flow cytometry (n=9-11). **(B-D, F)** Data shown as mean±SEM. **(C, D, F)** * p<0.05, ** p<0.01, *** p<0.001 and **** p<0.0001 by one-way ANOVA.

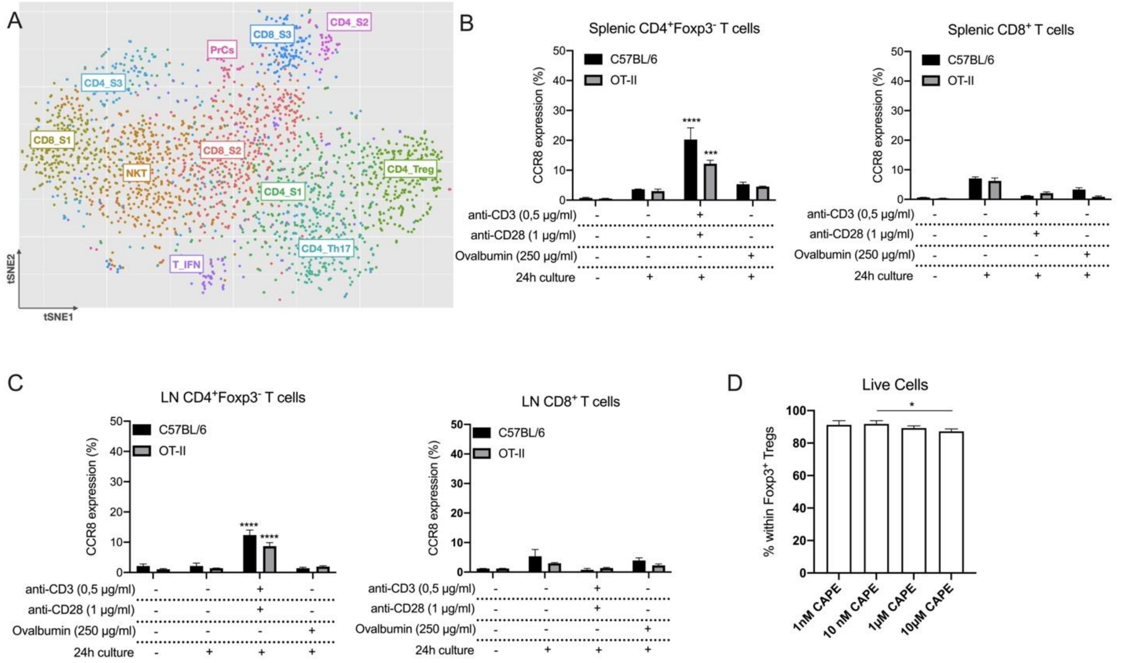

**Figure S3:** Additional data on CCR8 upregulation in response to TCR stimulation. **A.** tSNE plot based on regulon activity within the distinct T-cell subsets, determined using SCENIC. **B.** Percentage of CCR8$^+$ cells within the C57BL/6 and OT-II splenic Foxp3$^-$CD4$^+$ T cells or CD8$^+$ T cells after 24h of in vitro co-culture with TCR stimulants (anti-CD3 + anti-CD28) or Chicken ovalbumin (OVA) (n=3). **C.** Percentage of CCR8$^+$ cells within the C57BL/6 and OT-II LN Foxp3$^-$CD4$^+$ T cells or CD8$^+$ T cells after 24h of in vitro co-culture with TCR stimulants (anti-CD3 + anti-CD28) or Chicken ovalbumin (OVA) (n=3). **D.** Percentage of live Tregs after 24h of in vitro co-culture with TCR stimulants (anti-CD3 + anti-CD28) and distinct concentrations of CAPE (n=4). **(B-D)** Data shown as mean±SEM. **(B, C)** *** p<0.001 and **** p<0.0001 by one-way ANOVA where each condition was compared to prior to culture, **(D)** * p<0.05 by one-way ANOVA.

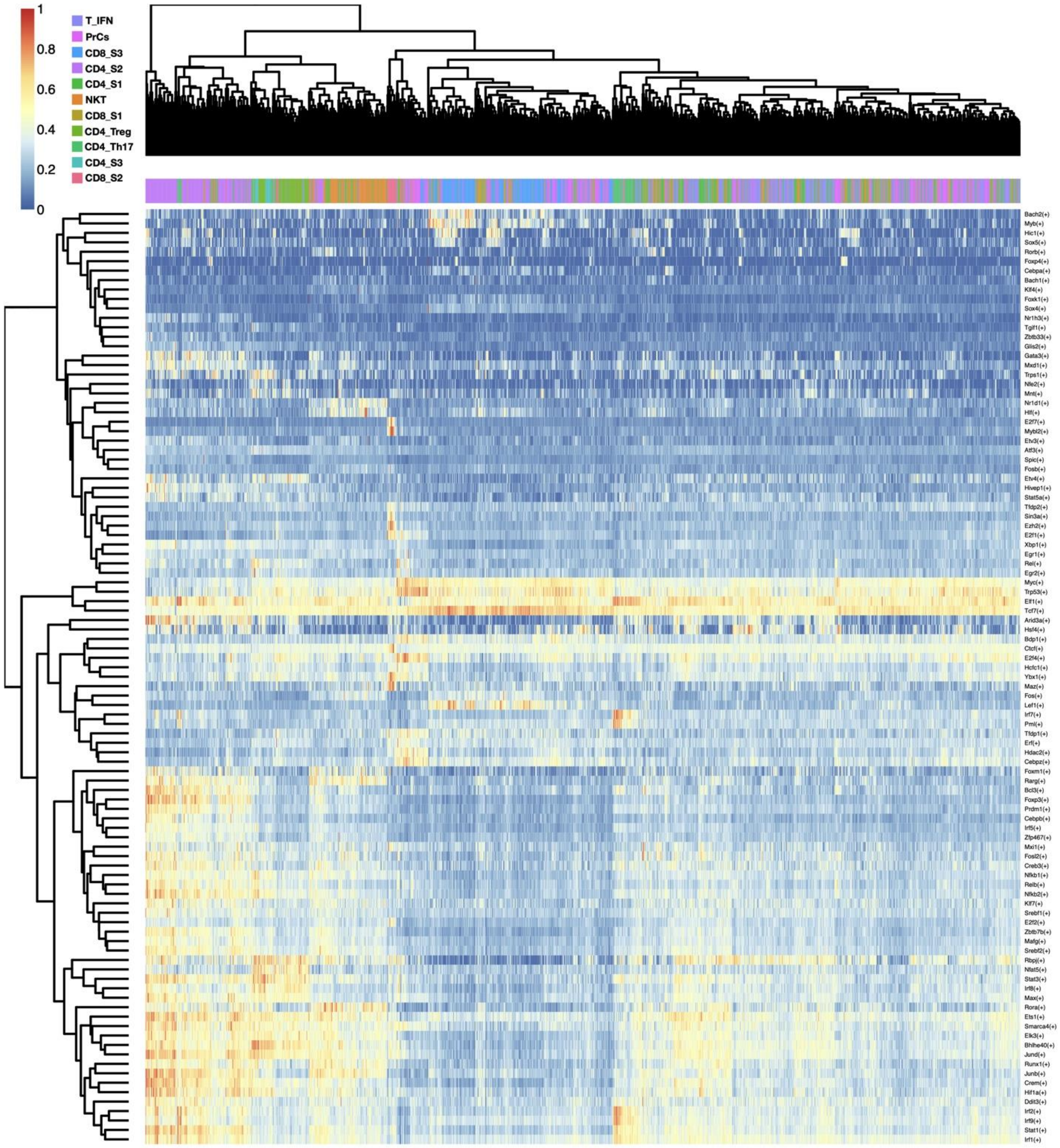


**Figure S4:** Regulon activity matrix as determined using SCENIC. Heatmap showing regulons that are correlated with any other regulon (abs(cor) > 0.30) and that are active in at least 1% of the NKT/T cells. For each regulon the level of activity within the distinct T-cell subsets is indicated with a color gradient.

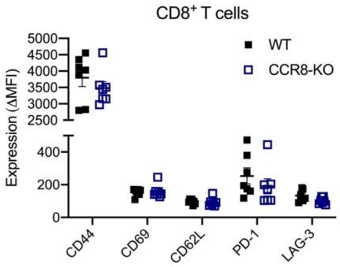


**Figure S5:** CD8$^+$ T cell activation status in CCR8-KO mice. Activation marker expression on CD8$^+$ T cells from LLC-OVA tumors grown in CCR8-KO versus WT littermate control mice (n=7), as determined via flow cytometry. Data shown as mean±SEM. Unpaired Student's t-test.

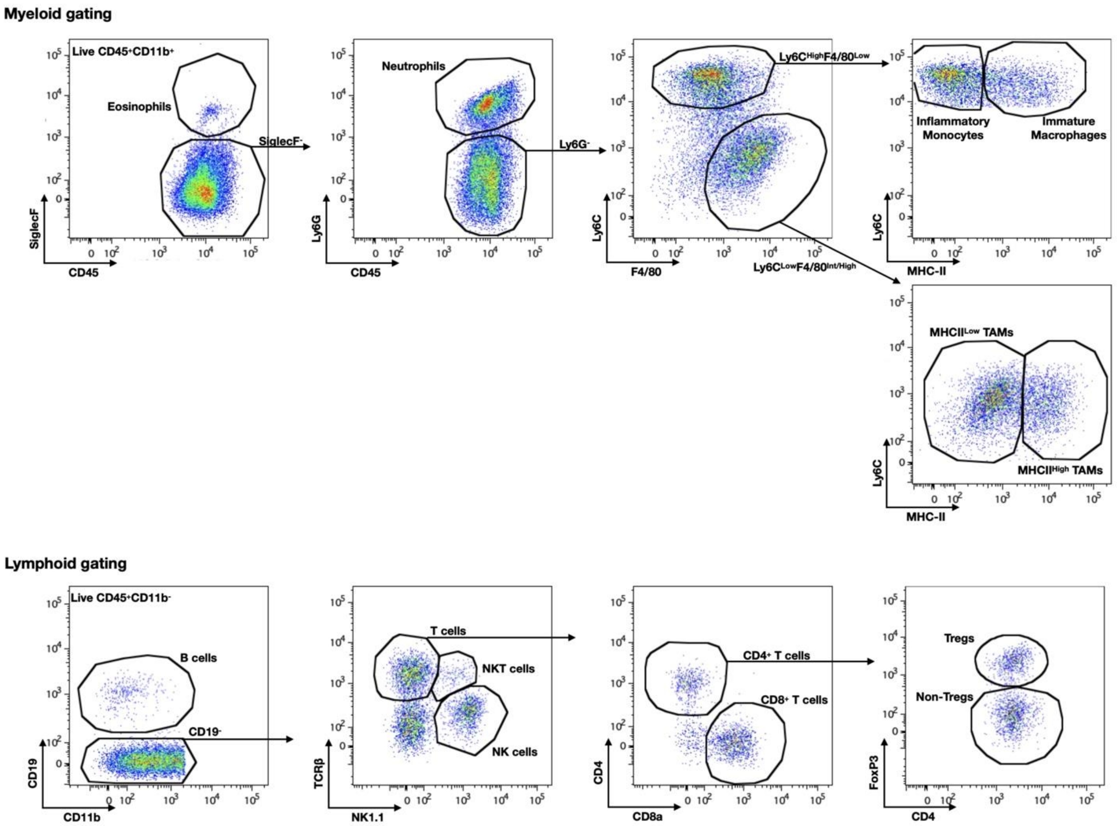


**Figure S6:** Myeloid and Lymphoid gating strategies. Representative overview of the gating strategies used in flow cytometry to identify the distinct tumor-infiltrating myeloid and lymphoid cell populations.

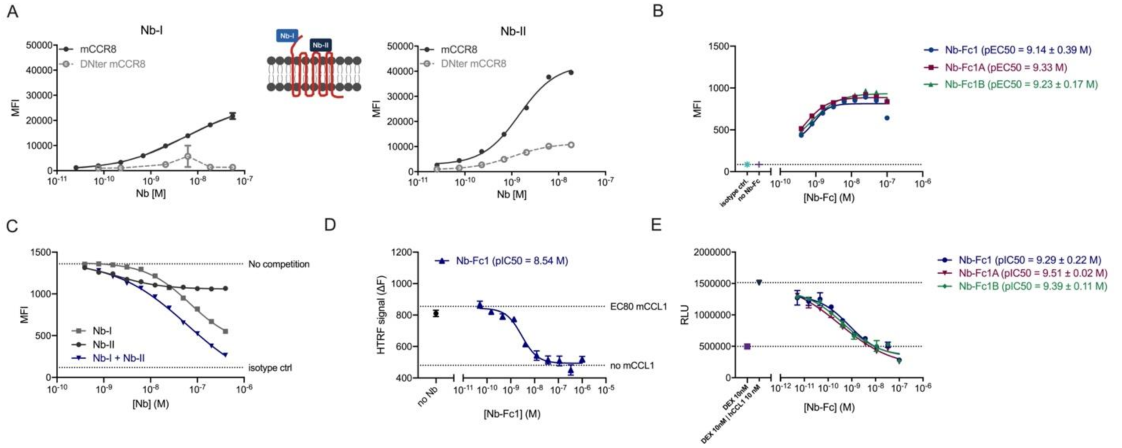


**Figure S7:** Nanobody binding profiles. **A.** Binding of increasing concentrations of Nb-I and Nb-II to HEK293 cells transfected with full length mCCR8 or CCR8 where the N-terminus has been deleted (DNter mCCR8). Measured using flow cytometry and shown as MFI. **B**. Binding characteristics of Nb-Fc1 (blue, n=6), Nb-Fc1A (red, n=1) and Nb-Fc1B (green, n=2) on CCR8-expressing BW5147 cells. Measured using flow cytometry and shown as MFI. **C**. Dilution series of monovalent Nb-I (grey), Nb-II (black) or a combination of both (blue) were competed against Nb-Fc1 on CCR8-expressing BW5147 cells. Measured using flow cytometry and shown as MFI. **D**. HTRF experiment to measure cAMP accumulation in CHO-K1 cells overexpressing mouse CCR8 upon mCCL1 treatment in the presence of Nb-Fc1 (n=1). **E**. Inhibition of the protective activity of the ligand CCL1 against dexamethasone-induced apoptosis on BW5147 cells by Nb-Fc1 (blue, n=9), Nb-Fc1A (red, n=2) and Nb-Fc1B (green, n=4). **(B-E)** Data shown as mean±SEM.

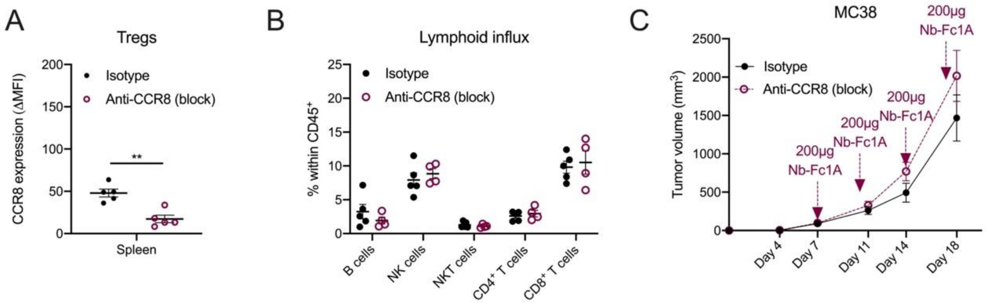


**Figure S8:** Additional data on CCR8 blockade. **A.** Relative expression of CCR8 (ΔMFI) by the Tregs in the spleen of isotype or anti-CCR8 (block) treated mice (n=5). **B.** Percentage of various lymphoid cell types within the CD45$^+$ population of D16 LLC-OVA tumors of isotype or anti-CCR8 (block)-treated mice (n=5). **C.** s.c. MC38 tumor growth in isotype or anti-CCR8 (block)-treated mice (n=9-10). **(A-C)** Data shown as mean±SEM. **(A, B)** ** p<0.01 by Student's t-test, **(C)** by two-way ANOVA with Holm-Sidak multiple comparisons test.

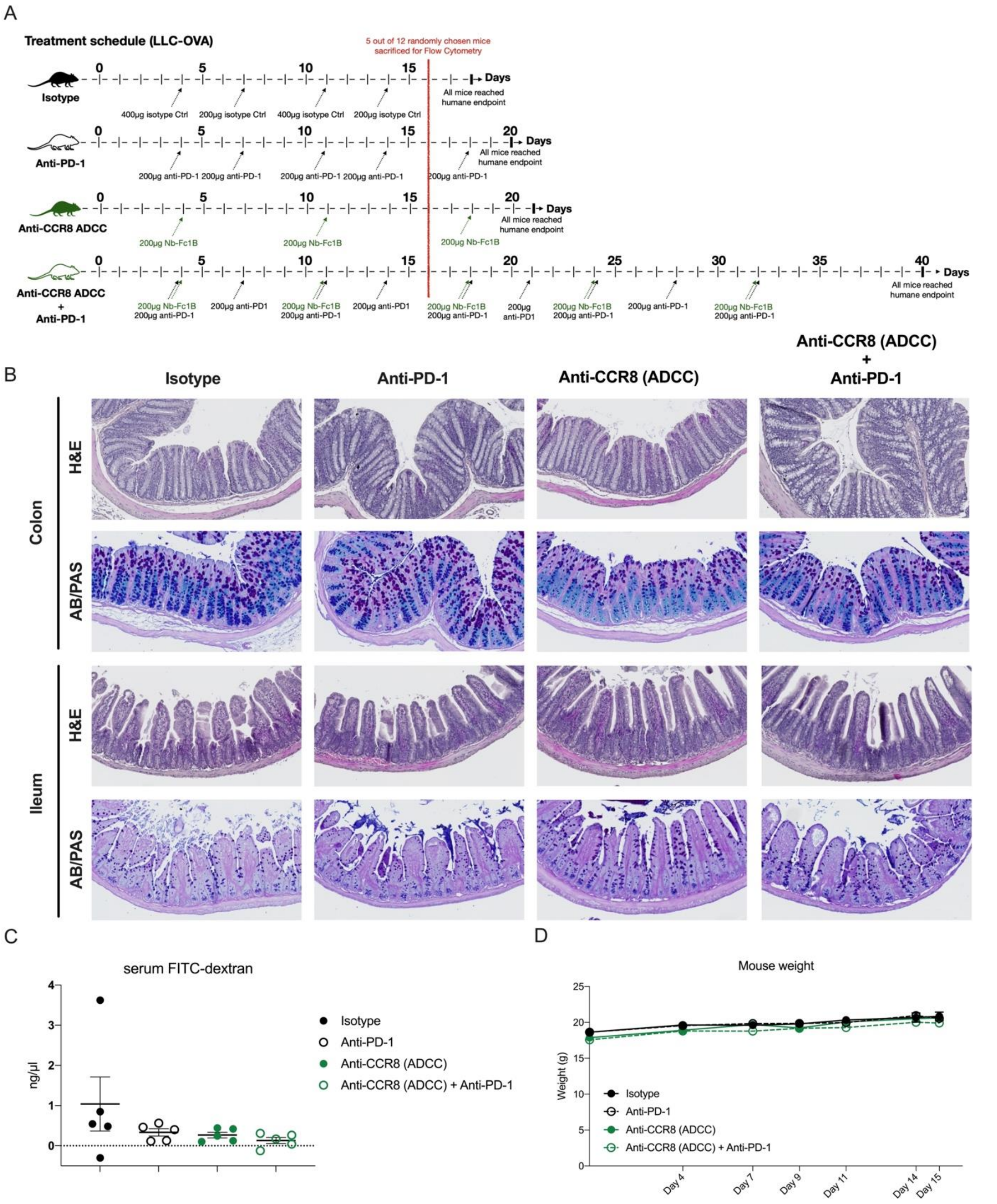

**Figure S9:** Therapeutic treatment of LLC-OVA tumor-bearing mice. **A.** Schematic overview of the treatment regimen with isotype, anti-PD-1, Nb-Fc1B (anti-CCR8 (ADCC)) and the combination of anti-PD-1 and Nb-Fc1B**. B.** Representative images of H&E and AB/PAS stained ileal and colonic sections of LLC-OVA tumor-bearing mice treated with with isotype, anti-PD-1 and/or Nb-Fc1B (anti-CCR8 (ADCC)). **C.** Serum FITC-dextran concentration of LLC-OVA tumor-bearing mice treated with with isotype, anti-PD-1 and/or Nb-Fc1B (anti-CCR8 (ADCC)) (n=5). **D**. Weights of LLC-OVA tumor-bearing mice treated with with isotype, anti-PD-1 and/or Nb-Fc1B (anti-CCR8 (ADCC)) (n=5). **(C,D)** Data shown as mean±SEM.

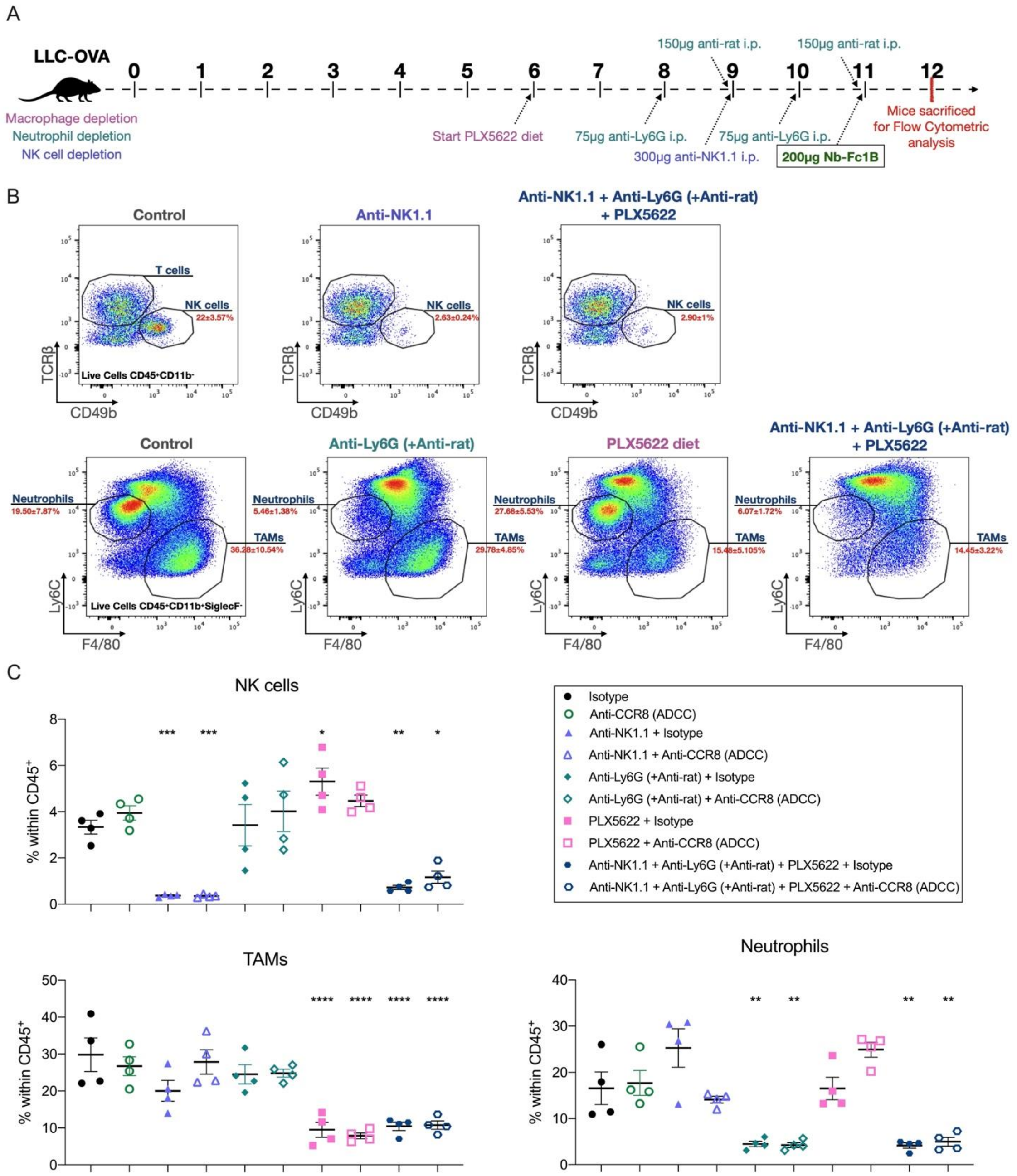


**Figure S10:** In vivo cell depletion. **A.** Schematic overview of the treatment regimen with Nb-Fc1B (anti-CCR8 (ADCC)), anti-NK1.1, anti-Ly6G (+Anti-rat) and PLX5622. **B.** Representative overview of the gating strategies used in flow cytometry to identify the NK cells, neutrophils and TAMs after their respective depletions. **C**. Percentage of NK cells, TAMs and neutrophils within the $CD45^+$ population of D12 LLC-OVA tumors treated with isotype control or Nb-Fc1B (anti-CCR8 (ADCC)) in combination with anti-NK1.1, Anti-Ly6G (+Anti-rat) and/or PLX5622 (n=4). **(B-C)** Data shown as mean±SEM. **(C)** * p<0.05, ** p<0.01, *** p<0.001 and **** p<0.0001 by one-way ANOVA where each condition was compared to the isotype control group.

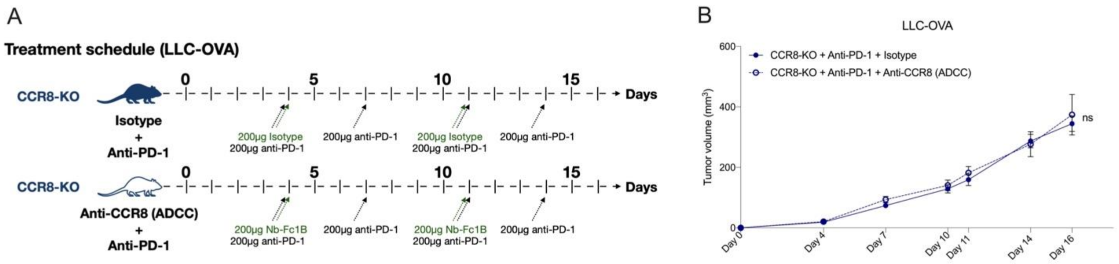


**Figure S11:** Anti-CCR8 (ADCC) therapy does not have a therapeutic effect in CCR8-KO mice. **A.** Schematic overview of the treatment regimen with anti-PD-1 and/or Nb-Fc1B (anti-CCR8 (ADCC)) in CCR8-KO mice. **B.** s.c. LLC-OVA tumor growth in CCR8-KO mice treated with anti-PD-1 (blue, closed circle) or the combination of anti-PD-1 and anti-CCR8 (ADCC) (blue, open circle) (n=8-9). Data shown as mean±SEM, significance determined by Two-way ANOVA with Holm-Sidak multiple comparisons test.

**Table S1:** amino acid sequences of the CCR8 fragments used for immunization/transfection.

| ID | Sequence |
|---|---|
| Mouse CCR8 (NP_031746) | MDYTMEPNVTMTDYYPDFFTAPCDAEFLLRGSMLYLAILYCVLFVLGLLGNSLVILVLVGCKKLRSITDIYLLNLAASDLLFVLSIPFQTHNLLDQWVFGTAMCKVVSGLYYIGFFSSMFFITLMSVDRYLAIVHAVYAIKVRTASVGTALSLTVWLAAVTATIPLMVFYQVASEDGMLQCFQFYEEQSLRWKLFTHFEINALGLLLPFAILLFCYVRILQQLRGCLNHNRTRAIKLVLTVVIVSLLFWVPFNVALFLTSLHDLHILDGCATRQRLALAIHVTEVISFTHCCVNPVIYAFIGEKFKKHLMDVFQKSCSHIFLYLGRQMPVGALERQLSSNQRSSHSSTLDDIL |
| Mouse CCR8 (delta16-3XHA) | MYPYDVPDYAAYPYDVPDYAGYPYDVPDYAFFTAPCDAEFLLRGSMLYLAILYCVLFVLGLLGNSLVILVLVGCKKLRSITDIYLLNLAASDLLFVLSIPFQTHNLLDQWVFGTAMCKVVSGLYYIGFFSSMFFITLMSVDRYLAIVHAVYAIKVRTASVGTALSLTVWLAAVTATIPLMVFYQVASEDGMLQCFQFYEEQSLRWKLFTHFEINALGLLLPFAILLFCYVRILQQLRGCLNHNRTRAIKLVLTVVIVSLLFWVPFNVALFLTSLHDLHILDGCATRQRLALAIHVTEVISFTHCCVNPVIYAFIGEKFKKHLMDVFQKSCSHIFLYLGRQMPVGALERQLSSNQRSSHSSTLDDIL |

**Table S2:** clinical characteristics of NSCLC and melanoma patients that participated in this study.

| Patient | | Sex | Smoking History | COPD | Histology mutations | Stage @ diagnosis | Treatment | Age Group |
|---|---|---|---|---|---|---|---|---|
| 1 | NSCLC | Female | Former | no | Squamous | IIIA | Platinum based chemotherapy | 65-70 |
| 2 | NSCLC | Male | current | yes | Adenocarcinoma | IVB | Platinum based chemotherapy | 70-75 |
| 3 | NSCLC | Male | Former | yes | Squamous | IVB | - | 65-70 |
| 4 | NSCLC | Male | Former | no | Squamous | IIB | Platinum based chemotherapy | 65-70 |
| 5 | NSCLC | Male | Former | no | Adenocarcinoma | IIIB | - | 85-90 |
| 6 | NSCLC | Male | Former | no | Adenocarcinoma | IIIA | Platinum based chemotherapy | 70-75 |
| 7 | NSCLC | Female | Former | no | Squamous | IB | - | 70-75 |
| 8 | NSCLC | Female | current | yes | Adenocarcinoma | IIB | Platinum based chemotherapy | 55-60 |
| 9 | NSCLC | Male | Former | yes | Adenocarcinoma | IA | - | 65-70 |
| 10 | Melanoma | Female | - | - | BRAF mutant | IV-M1d | Ipilimumab Pembrolizumab Dafranib Trametinib Chemo (temozolomide) | 50-55 |
| 11 | Melanoma | Female | - | - | NRAS mutant | IV-M1c | Ipilimumab Pembrolizumab Chemo (temozolomide) | 70-75 |

**Table S3:** antibodies used in the flow cytometric analysis of both mouse and human samples.

| Target | Fluorophore | Species Reactivity | Provider | Clone |
|---|---|---|---|---|
| CD4 | FITC | Mouse | eBioscience | GK1.5 |
| MHCII | Alexa Fluor 488 | Mouse | BioLegend | M5/114.15.2 |
| CD11b | Alexa Fluor 488 | Mouse | BD Biosciences | M1/70 |
| CD44 | FITC | Mouse | eBioscience | IM7 |
| TCRβ | FITC | Mouse | eBioscience | H57-597 |
| CD25 | FITC | Mouse | BD Biosciences | 7D4 |
| CD49b | FITC | Mouse | eBioscience | HMa2 |
| CD8a | PE | Mouse | BD Biosciences | 53-6.7 |
| Siglec-F | PE | Mouse | BD Biosciences | E50-2440 |
| PD-1 (CD279) | PE | Mouse | BD Biosciences | J43 |
| CCR8 (CD196) | PE | Mouse | BioLegend | SA214G2 |
| NK1.1 | PerCP/Cyanine5.5 | Mouse | eBioscience | PK136 |
| Ly-6G | PerCP/Cyanine5.5 | Mouse | BioLegend | 1A8 |
| CD11c | PerCP/Cyanine5.5 | Mouse | eBioscience | N418 |
| CD62L | PerCP/Cyanine5.5 | Mouse | BioLegend | MEL-14 |
| Foxp3 | PerCP/Cyanine5.5 | Mouse, Bovine, Dog, Cat, Pig, Rat | eBioscience | FJK-16s |
| CD11b | PE/Cyanine7 | Human, Mouse | BioLegend | M1/70 |
| CD24 | PE/Cyanine7 | Mouse | BD Biosciences | M1/69 |
| OX-40 (CD134) | PE/Cyanine7 | Mouse | BioLegend | OX-86 |
| TCRβ | APC | Mouse | eBioscience | H57-597 |
| Ly-6C | Alexa Fluor 647 | Mouse | BioLegend | HK1.4 |
| CD64 | APC | Mouse | BioLegend | X54-5/7.1 |
| Neuropilin-1 (CD304) | APC | Mouse | BioLegend | 3E12 |
| CD45 | APC/Cyanine7 | Mouse | BioLegend | 30-F11 |
| CD19 | Brilliant Violet 421 | Mouse | BD Biosciences | 1D3 |
| F4/80 | Brilliant Violet 421 | Mouse | BioLegend | EMR1, Ly71 |
| MHCII (I-A/I-E) | Brilliant Violet 421 | Mouse | BioLegend | M5/114.15.2 |
| CD8a | Brilliant Violet 421 | Mouse | BD Biosciences | 53-6.7 |
| CD4 | Brilliant Violet 421 | Mouse | BD Biosciences | GK1.5 |
| CD69 | FITC | Mouse | BioLegend | H1.2F3 |
| GARP | APC | Mouse | eBioscience | YGIC86 |
| Helios | FITC | Mouse | eBioscience | 22F6 |
| LAG-3 (CD223) | APC | Mouse | BioLegend | C9B7W |
| KLRG1 | APC | Human, Mouse | BioLegend | 2F1/KLRG1 |
| CD3 | PerCP/Cyanine5.5 | Human | BioLegend | HIT3a |
| CD4 | APC/Cyanine7 | Human, Chimpanzee, Cynomolgus, Rhesus | BioLegend | OKT4 |
| CD127 | FITC | Human, African Green, Baboon, Cynomolgus, Rhesus | BioLegend | A019D5 |
| CD25 | PE/Cyanine7 | Human, Baboon, Chimpanzee, Pigtailed Macaque, Rhesus | BioLegend | BC96 |
| CCR8 (CD198) | PE | Human | BioLegend | L263G8 |
